# Pharmacokinetics and Tolerability of Inhaled Umeclidinium and Vilanterol Alone and in Combination in Healthy Chinese Subjects: A Randomized, Open-Label, Crossover Trial

**DOI:** 10.1371/journal.pone.0121264

**Published:** 2015-03-27

**Authors:** Chaoying Hu, Jingying Jia, Kelly Dong, Linda Luo, Kai Wu, Rashmi Mehta, Jack Peng, Yan Ren, Annette Gross, Hui Yu

**Affiliations:** 1 Phase I Clinical Research Unit, Shanghai Xuhui Central Hospital, Shanghai, China; 2 PTS China, GSK R&D, Shanghai, China; 3 Statistics, Program and Data Management, GSK R&D, Shanghai, China; 4 Clinical Pharmacology Modelling & Simulation, GSK R&D, Shanghai, China; 5 Clinical Pharmacology Modelling & Simulation, GSK R&D, Research Triangle Park, NC, United States of America; 6 Clinical Pharmacology Modelling & Simulation, GSK R&D, Ermington, Australia; 7 Clinical Medicine Development, GSK R&D, Shanghai, China; National Cancer Centre, SINGAPORE

## Abstract

Inhaled umeclidinium (UMEC) and the combination of inhaled UMEC with vilanterol (UMEC/VI) are approved maintenance treatments for chronic obstructive pulmonary disease in the US and EU. This was a randomized, open-label, three-period crossover, single- and repeat-dose study to assess the pharmacokinetics (PK), safety, and tolerability of inhaled UMEC/VI 62.5/25 μg (delivering 55/22 μg) and UMEC/VI 125/25 μg (delivering 113/22 μg) compared with their monotherapy components (UMEC 62.5 μg, UMEC 125 μg and, VI 25 μg [delivering 55, 113, and 22 μg, respectively]) in healthy Chinese subjects (n=20). UMEC and VI were rapidly absorbed following single and repeat dosing (time to maximum plasma concentration [t_max_]: UMEC = 5 min; VI = 5 min). The median t_last_ was 2–4 h for UMEC and 1–2 h for VI following single doses of UMEC/VI and UMEC monotherapy (both doses). UMEC reached steady-state prior to Day 10; steady-state for VI could not be assessed. UMEC accumulation following repeat dosing was 11–34% based on C_max_ and 19–59% based on area under the concentration-time curve from time zero to 2 h (AUC_(0-2)_). VI accumulation following repeat dosing was 25–66% based on C_max_ and 17–43% based on AUC_(0-2)_. The evidence was not sufficient to suggest that systemic exposure was substantially different between UMEC/VI combination therapy and the constituent monotherapies following single or repeat dosing. Following both single- and repeat-dose administration, the inter-subject coefficient of variation for all UMEC PK parameter estimates ranged from 12% to 165% for all treatments, indicating a wide range of variability in inhaled PK parameters. Twelve subjects experienced ≥1 adverse event (AE). Six subjects experienced ≥1 treatment-related AE; the most commonly reported treatment-related AE was chest discomfort (n=3 [15%]). No clinically important changes in vital signs or electrocardiogram parameters were reported. These data suggest that single- and repeat-dose administration of UMEC/VI combination therapy in healthy Chinese subjects did not result in substantial differences in systemic exposure compared with UMEC and VI as monotherapies.

**Trial Registration:**

Clinicaltrials.gov NCT01899638 NCT01899638

## Introduction

Chronic obstructive pulmonary disease (COPD) is a progressive condition characterized by airflow obstruction and reduced maximum expiratory flow, which can lead to restricted activity and poor quality of life.[1] Long-acting muscarinic antagonists (LAMAs) and long-acting β_2_-agonists (LABAs) feature prominently in the recommended management strategy for the treatment of patients with COPD.[2,3] The distinct and complementary mechanisms of action of these two drug classes presents the opportunity for combination therapy, which could provide additional therapeutic benefits over monotherapy.[4]

The inhaled LAMA umeclidinium (UMEC, GSK, London, UK) and the combination of UMEC with the inhaled LABA vilanterol (UMEC/VI; GSK, London, UK) are approved maintenance treatments for COPD in the US and EU.[5,6] Studies have shown that once-daily UMEC and VI administered alone or in combination can provide clinically significant, sustained bronchodilation over 24 hours (h), with similar adverse event (AE) profiles to placebo.[7–11]

For inhaled COPD treatments, efficacy is considered to relate to the drug present in the lung. However, drug absorbed into the systemic circulation may theoretically elicit additional pharmacological effects which could contribute to the safety and tolerability profile of the drug. While pharmacokinetic (PK) studies in Western populations have shown that UMEC and VI exhibit low systemic exposure when administered via oral inhalation, PK data in Chinese subjects have not been reported.[12] As inter-ethnic differences in PK have been reported for some drugs, including following oral inhalation, characterization of inhaled UMEC and VI PK in Chinese subjects following oral inhalation is warranted.[13,14] Therefore, the PK, safety, and tolerability of single and repeat doses of orally inhaled UMEC and VI were assessed in this study, both in combination and as monotherapies, in healthy Chinese subjects.

## Materials and Methods

### Study design and treatment

This was a single-center, randomized, open-label, three-period crossover, balanced, incomplete block study in healthy Chinese subjects (Clinicaltrials.gov: NCT01899638; GSK study number: DB2115380). The study was performed at the Phase 1 Clinical Research Unit, Shanghai Xuhui Central Hospital, between May 20, 2013 and July 25, 2013.

Each subject was randomized to a sequence of three of five treatments in accordance with a pre-specified randomization schedule: UMEC/VI 62.5/25 μg (delivering 55/22 μg), UMEC/VI 125/25 μg (delivering 113/22 μg), UMEC 62.5 μg (delivering 55 μg), UMEC 125 μg (delivering 113 μg), or VI 25 μg (delivering 22 μg). Each treatment was administered once daily for 10 days via an ELLIPTA dry powder inhaler, with a minimum washout period of 7–14 days between dosing periods. PK data following the first dose on Day 1 represented single-dose PK, and PK data collected on Day 10 represented repeat-dose PK. Each subject underwent screening within 21 days of receiving their first dose of study medication and had a follow-up visit 7–10 days after the last dose.

### Subjects

Eligible subjects were healthy males and females (1:1 ratio) of Chinese heritage that were: 18–45 years of age; non-smokers who had a smoking history of ≤10 pack—years and had not used any tobacco products in the 6-month period preceding the screening visit, had a body weight ≥50 kg, a body mass index (BMI) of 19–24 kg/m^2^, and normal systolic blood pressure (BP; 90–139 mmHg) and diastolic BP (60–89 mmHg); had no relevant abnormality on 12-lead electrocardiogram (ECG), QTcF interval <450 msec, and no significant clinical abnormalities. A complete list of inclusion and exclusion criteria is provided in S1 Supporting Information.

All subjects provided written informed consent. The study was conducted in accordance with International Conference on Harmonisation of Technical Requirements for Registration of Pharmaceuticals for Human Use Good Clinical Practice guidelines, all applicable subject privacy requirements, and the 2008 Declaration of Helsinki.[15] The study protocol and informed consent form were approved by the independent ethics committee of Shanghai Xuhui Central Hospital, Shanghai, China. The protocol for this trial and supporting CONSORT checklist are available as supporting information; see S1 Protocol and S1 Checklist.

Blood samples for plasma PK analyses were collected on Day 1 and Days 6–12 (pre-dose only on Days 6–9) in each treatment period. On Day 1, UMEC and VI plasma concentrations were determined pre-dose and at 5, 15, and 30 minutes (min), and 1, 2, 4, 6, and 8 h post-dose. On Day 10 (i.e. up to Day 12) UMEC and VI plasma concentrations were determined pre-dose and at 5, 15, and 30 min and 1, 2, 4, 6, 8, 12, 24, and 48 h post-dose. UMEC and VI plasma concentrations were measured at Wuxi App Tec (Wuxi AppTec Co. Ltd, Shanghai, China) by a validated bioanalytical method using solid phase extraction and high-performance liquid chromatography/mass spectrometry. The calibration curves ranged from 10.0 pg/mL to 2000 pg/mL and 10.0 pg/mL to 1000 pg/mL for UMEC and VI, respectively. Five quality control samples of different concentrations including samples at lower limit of quantitation (LLOQ) level were analyzed and all acceptable analytical runs met predefined acceptance criteria. For all quality control levels, the within-run precision and accuracy were: 1.0–15.0% and -9.3–10.0% for UMEC, 0.9–10.7%, and -3.7–7.0% for VI, respectively. The between-run precision and accuracy were: 2.7–11.6% and -2.3–4.0% for UMEC, 3.3–9.0% and -2.3–6.0% for VI, respectively.

A cross-validation between Unilabs (York Bioanalytical Solutions, York, UK) and Wuxi AppTec Co. Ltd, (Shanghai, China) was performed to compare the accuracy of measurements from the two different laboratories to allow data comparison between studies. Acceptable accuracy between two laboratories was defined as ≤±20.0% for each sample.

### Pharmacokinetic analyses

Plasma concentration—time data for UMEC and VI were analyzed using non-compartmental; extravascular Model 200–202 of Phoenix WinNonlin Professional Edition version 6.2.1 (Pharsight Corporation, St. Louis, MO, USA). Calculations were based on the actual sampling times recorded during the study.

The derived PK parameters summarized for UMEC and VI on Day 1 and Day 10 included: maximum plasma concentration (C_max_); time to C_max_ (t_max_); time of last quantifiable concentration (t_last_); area under the plasma concentration—time curve from time zero (pre-dose) to common quantifiable time points (AUC_(0–t’)_), where t’ was 2 h. Area under the concentration—time curve over a dosage interval from time zero (pre-dose) extrapolated to infinite time (AUC_(0–∞)_) was assessed for UMEC and VI only at Day 1. Area under the concentration—time curve at steady-state AUC_(0–τ)_ was calculated for UMEC at Day 10 only, where the dosing interval (τ) was 24 h. Area under the plasma concentration—time curve from time zero to t_last_ (AUC_0–t_) was assessed for UMEC and VI at Day 1 and Day 10. AUC_(0–t’)_ and AUC_(0–t)_ were determined using the linear trapezoidal rule for increasing concentrations and the logarithmic trapezoidal rule for decreasing concentrations.

### Safety analyses

AEs and serious AEs (SAEs) were collected from the start of dosing, throughout the treatment periods, and up to the follow-up visit (which fell either during the washout period [where an additional treatment period was to follow] or post-last dose). The washout period of 7–14 days between treatment periods was adequate to ensure potential AEs that developed in this time were reported and resolved before the start of the following treatment period. Vital signs, including BP (systolic and diastolic) and heart rate (HR) were measured at screening, on each day of each treatment period, and at follow-up. Twelve-lead ECGs were measured on Day 1 and Day 10; maximum 0–4 h QTcF and maximum 0–4 h HR as well as 0–4 h weighted mean QTcF and 0–4 h weighted mean HR were determined. Clinical laboratory evaluations, including clinical chemistry, hematology, and urinalysis were conducted at screening, during treatment, and at follow-up.

### Statistical methods

No formal sample size calculations were performed. Recommendations from the China Food and Drug Administration indicate that a sample size of 8–12 evaluable subjects in each arm is required for PK evaluation of a pharmacological agent. Using the three-way crossover, balanced, incomplete block design presented in S1 Table, 20 subjects randomized to each of the 20 treatment regimens would provide 12 subjects for each of the five treatment arms (A—E). In addition, treatments are balanced evenly across the three treatment periods. As such, 20 subjects were enrolled to achieve an estimated 10–12 evaluable subjects in each treatment arm.

Two analysis populations were defined: the PK population comprised all subjects who received at least one dose of study medication and for whom a PK sample was obtained and analyzed. The safety population included all subjects who received at least one dose of study medication.

As the study was not powered to detect specific differences in treatment effects, no formal statistical comparisons were prospectively planned or performed and the results are presented descriptively. Parameters are reported as geometric means and corresponding 95% confidence intervals (CIs), except t_max_ and t_last_, which are reported as median (range). The ratio of the combination therapy to the monotherapies was also calculated, with 90% CIs.

Steady-state according to treatment group was assessed for UMEC by statistical analysis of log-transformed trough concentrations from Day 6 to Day 10, and the 24-h post-dose concentration on Day 10 (Day 11). Steady-state was defined as a slope estimate (90% CI) of the linear regression of trough concentration versus time between 0.91 and 1.10 for back-transformed values (-0.0943–0.0953 of log_e_).

Accumulation of UMEC and VI was assessed separately using log-transformed data from Day 1 and Day 10. Subjects with plasma concentrations below the LLOQ were excluded from statistical analyses.

## Results

### Baseline characteristics and subject disposition

**Fig 1:**
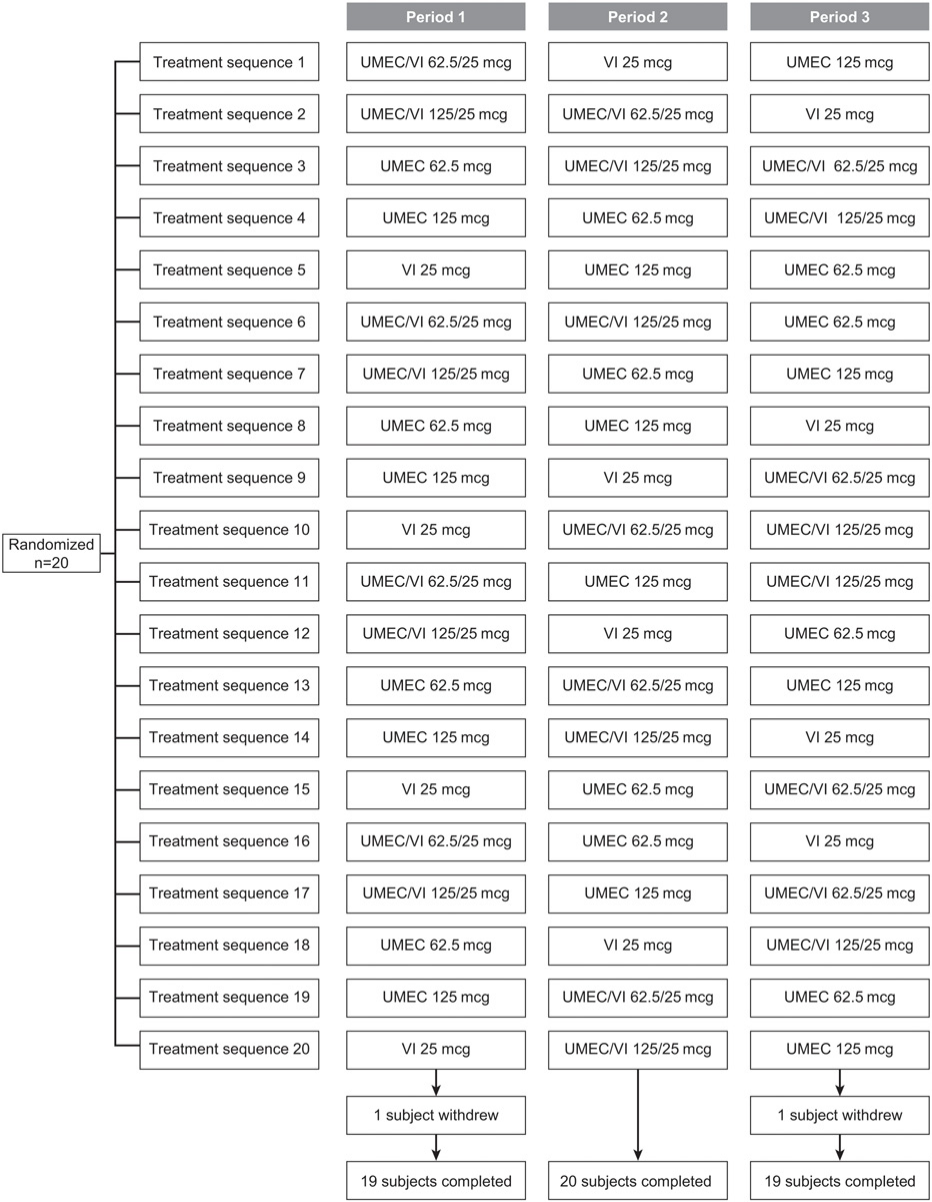
Subject disposition. UMEC, umeclidinium; VI, vilanterol.

Twenty healthy subjects (10 male and 10 female) of East Asian heritage and resident in China were enrolled. The subjects had a mean age of 26.2 years, a mean BMI of 21.7 kg/m^2^, and mean body weight of 59.3 kg (Table 1). The majority of subjects (90%) completed the study. Two subjects (10%) withdrew from the study due to personal reasons; one subject (assigned treatment sequence: VI 25 μg, UMEC 62.5 μg, UMEC/VI 62.5/25 μg) withdrew in Treatment Period 3 after 6 days of treatment with UMEC/VI 62.5/25 μg and the other subject (assigned treatment sequence: UMEC 62.5 μg, VI 25 μg, UMEC/VI 125/25 μg) withdrew in Treatment Period 1 after 10 days of treatment with UMEC 62.5 μg (Fig. 1).

**Table 1:** Baseline demographic characteristics.

Characteristic	Total (N = 20)
Age, years	
Mean (SD)	26.2 (5.00)
Median (Range)	25.0 (18–36)
Sex, n (%)	
Female	10 (50)
Male	10 (50)
Height, cm	
Mean (SD)	165.14 (4.89)
Median (Range)	164.80 (158.2–175.7)
Weight, kg	
Mean (SD)	59.31 (5.86)
Median (Range)	60.45 (50.6–70.4)
Body mass index, kg/m^2^	
Mean (SD)	21.71 (1.47)
Median (Range)	21.83 (19.4–24.0)
Race, n (%)	
Asian—East Asian Heritage	20 (100)

SD, standard deviation

### Pharmacokinetics

#### UMEC

**Fig 2:**
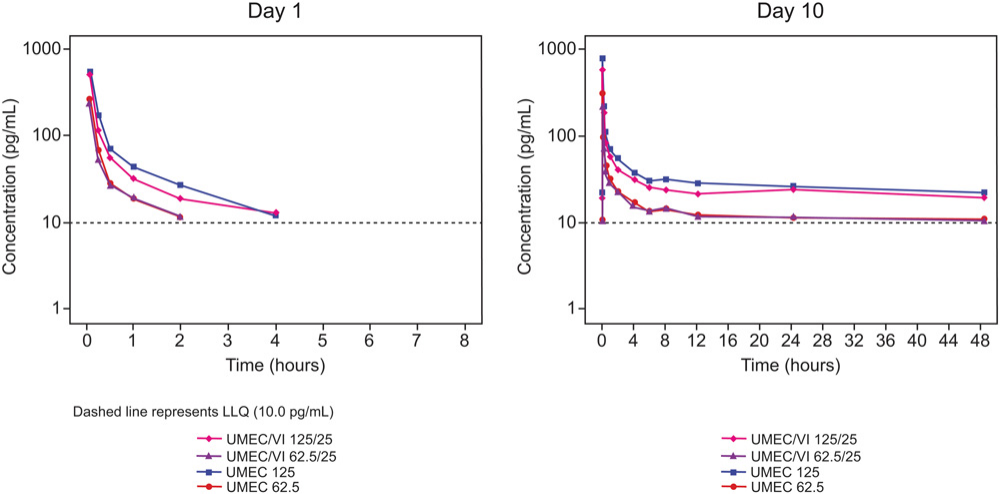
Median UMEC plasma concentration—time profiles on Day 1 and Day 10. LLOQ, lower limit of quantification; PK, pharmacokinetic; UMEC, umeclidinium; VI, vilanterol.

UMEC was rapidly absorbed with a median t_max_ of 0.08 h (5 min) following both single- and repeat-dose administration of UMEC/VI and UMEC monotherapy (Fig. 2; Table 2).

**Table 2:** UMEC PK parameters following single- and repeat-dose administration of UMEC/VI and UMEC monotherapy (PK population).

Parameter	UMEC/VI 62.5/25 μg (N = 12)	UMEC/VI 125/25 μg (N = 11)	UMEC 62.5 μg (N = 12)		UMEC 125 μg (N = 12)	Ratio of adjusted geometric means (90% CI)^#^	
						**UMEC/VI 62.5/25 μg vs UMEC 62.5 μg**	**UMEC/VI 125/25 μg vs UMEC 125 μg**
**Single-dose administration study, Day 1**							
C_max_, pg/mL	214 (182, 252)	471 (380, 584)		234 (188, 290)	569 (448, 721)	0.97 (0.79, 1.19)	0.90 (0.73, 1.10)
t_max_, h*	0.08 (0.08–0.08)	0.08 (0.08–0.10)		0.08 (0.08–0.10)	0.08 (0.08–0.10)	N/A	N/A
t_last_, h*	2.0 (1.0–4.0)	4.0 (2.0–8.0)		2.0 (0.3–8.0)	4.0 (2.0–8.0)	N/A	N/A
AUC_(0–2)_, pg.h/mL	49.2 (32.2, 75.2)	134 (114, 159)		53.3 (33.7, 84.5)	161 (132, 198)	0.89 (0.74, 1.06)	0.88 (0.74, 1.04)
AUC_(0-∞)_, pg.h/mL	78.2 (42.8, 143)	169 (106, 270)		79.9 (53.6, 119)	229 (167, 314)	N/A	0.78 (0.57, 1.07)
**Repeat-dose administration study, Day 10**							
C_max_, pg/mL^†^	245 (193, 311)	548 (450, 668)		258 (178, 376)	759 (598, 965)	0.88 (0.62, 1.24)	0.77 (0.66, 0.90)
t_max_, h* ^†^	0.08 (0.08–0.10)	0.08 (0.08–0.10)		0.08 (0.08–0.10)	0.08 (0.08–0.10)	N/A	N/A
t_last_, h* ^†^	48.0 (2.0–48.1)	48.0 (48.0–48.0)		48.0 (2.0–48.0)	48.0 (48.0–48.0)	N/A	N/A
AUC_(02)_, pg/mL^†^	89.1 (73.8, 108)	192 (168, 220)		93.2 (70.7, 123)	257 (213, 310)	0.91 (0.82, 1.02)	0.79 (0.75, 0.83)
AUC_(0τ)_, pg.h/mL^†^	304 (204, 453)	720 (634, 818)		298 (178, 499)	910 (766, 1080)	0.89 (0.75, 1.05)	0.83 (0.75, 0.93)

Data shown as geometric mean (95% CI) unless otherwise indicated.

^#^A mixed model fitted with period, treatment fixed effect and subjects as the random effect was used in this analysis for Day 1 and Day 10 separately. Log_e_-transformed AUC and C_max_ values were used in the model. The treatment ratios were calculated by back-transforming the difference between the adjusted means.

*Data shown as median (range);

^†^UMEC/VI 62.5/25 μg, n = 11.

AUC_(0–2)_, area under the concentration—time curve from time zero (pre-dose) to 2 hours; AUC_(0–∞)_, area under the concentration—time curve from time zero (pre-dose) extrapolated to infinite time; AUC_(0–τ)_, area under the concentration-time curve over the dosing interval; C_max_, maximum observed concentration; CI, confidence interval; N/A, not available (insufficient data to derive measure); PK, pharmacokinetic; t_last_, time of last quantifiable concentration; t_max_, time of occurrence of C_max_; UMEC, umeclidinium; VI, vilanterol.

The median t_last_ was 2–4 h after single doses of UMEC/VI and UMEC monotherapy (both doses), indicating rapid disposition of drug from systemic circulation. UMEC elimination half-life could not be measured in many cases as a large number of later samples had UMEC plasma concentrations below the LLOQ (138/874 samples [15.8%]). In comparison, the median t_last_ was 48 h following repeat dosing. UMEC displayed a rapid initial decline in plasma concentrations, with a terminal elimination phase after 6–8 h.

UMEC steady-state was attained prior to Day 10 for all treatments; slope estimates and 90% CIs for linear regression of measurable trough concentrations all were within -0.0943–0.0953 (S2 Table). Across all administered doses, UMEC accumulation was 11–34% based on C_max_ and 19–59% based on AUC_(0–2)_ (S3 Table).

Following both single and repeat dosing, C_max_ was lower when comparing UMEC/VI with UMEC monotherapy (single dosing, 10–13% lower; repeat dosing, 12–23% lower [Table 2]). Similarly, AUC_(0–τ)_ was also lower following repeat dosing, for both doses of UMEC/VI compared with UMEC monotherapy (11–17% lower). However, the 90% CI for the ratio of adjusted geometric means included 1.00 for all single-dose PK parameters and for the majority of the repeat-dose PK parameters (Table 2).

Although dose-proportionality was not formally assessed, UMEC C_max_ was more than 2-fold higher for UMEC/VI 125/25 μg compared with UMEC/VI 62.5/25 μg, following both single and repeat doses (Table 2). Similarly, AUC_(0–)_ was more than 2-fold higher for UMEC/VI 125/25 μg compared with UMEC/VI 62.5/25 μg following repeat dosing.

Following both single- and repeat-dose administration, the inter-subject coefficient of variation for all UMEC PK parameter estimates was 18–165% for UMEC/VI 62.5/25 μg and UMEC 62.5 μg, and 12–67% for UMEC/VI 125/25 μg and UMEC 125 μg.

**Fig 3:**
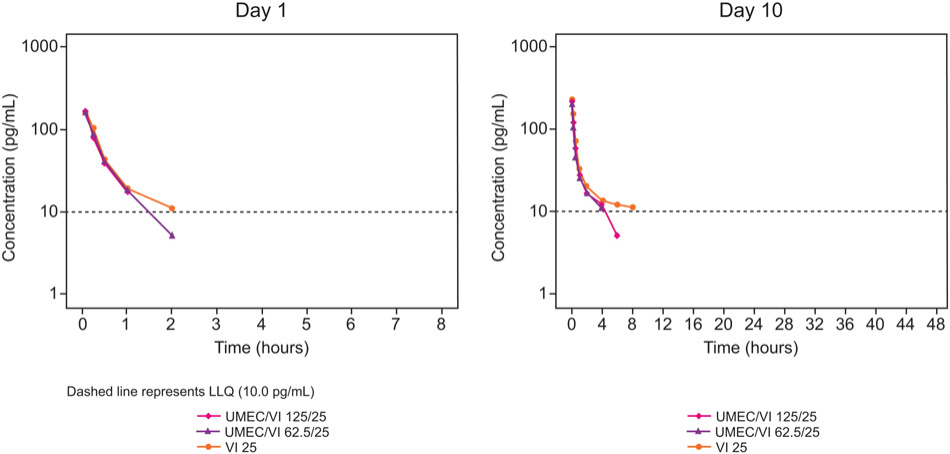
Median VI plasma concentration-time profiles on Day 1 and Day 10. LLOQ, lower limit of quantification; PK, pharmacokinetic; UMEC, umeclidinium; VI, vilanterol.

**VI**. VI was rapidly absorbed with a median t_max_ of 0.08 h (5 min) following both single- and repeat-dose administration of UMEC/VI and VI monotherapy (Fig. 3; Table 3). The median t_last_ was 1–2 h for VI following single doses of UMEC/VI and also following UMEC monotherapy (both doses). Full characterization of the VI PK profile was not possible as a high proportion of VI plasma samples were below the LLOQ (248/634 samples [39.1%]).

**Table 3:** VI PK parameters following single- and repeat dose administration of UMEC/VI and VI monotherapy (PK population).

Parameter	UMEC/VI 62.5/25 μg (N = 12)	UMEC/VI 125/25 μg (N = 11)	VI 25 μg (N = 11)	Ratio of adjusted geometric means (90% CI)^#^	
				**UMEC/VI 62.5/25 μg vs VI 25 μg**	**UMEC/VI 125/25 μg vs VI 25 μg**
**Single-dose administration study, Day 1**					
C_max_, pg/mL	163.8 (142.0, 189.1)	127.2 (71.1, 227.5)	169.5 (143.7, 200.1)	0.96 (0.83, 1.12)	1.04 (0.71, 1.50)
t_max_, h*	0.08 (0.08–0.08)	0.08 (0.08–0.10)	0.08 (0.08–0.10)	N/A	N/A
t_last_, h*	1.5 (1.0–6.0)	1.0 (0.1–2.0)	2.0 (1.0–2.0)	N/A	N/A
AUC_(02)_, pg.h/mL	49.1 (33.7, 71.6)	43.9 (31.2, 61.8)	62.0 (43.8, 87.7)	1.14 (0.93, 1.40)	0.89 (0.74, 1.06)
AUC_(0∞)_, pg.h/mL	68.9 (56.4, 84.1)	71.7 (57.5, 89.4)	84.3 (70.8, 100)	0.81 (0.66, 0.99)	0.87 (0.69, 1.09)
**Repeat-dose administration study, Day 10**					
C_max_, pg/mL^†^	205 (171, 245)	211 (174, 256)	239 (195, 292)	0.86 (0.73, 1.02)	0.89 (0.73, 1.09)
t_max_, h^†^	0.08 (0.08–0.10)	0.08 (0.08–0.10)	0.08 (0.08–0.08)	N/A	N/A
t_last_, h^†^	4.0 (1.0–12.0)	6.0 (2.0–8.0)	8.0 (6.0–12.1)	N/A	N/A
t_½_, h^‡^	1.84 (0.93, 3.65)	1.99 (0.75, 5.29)	N/A	N/A	N/A
AUC_(02)_, pg.h/mL^†^	84.5 (63.7, 112)	95.9 (80.9, 114)	119 (98.3, 145)	0.78 (0.66, 0.92)	0.81 (0.68, 0.97)
AUC_(0–t)_, pg.h/mL^†^	118 (86, 163)	136 (104, 177)	214 (172, 268)	N/A	N/A

Data shown as geometric mean (95% CI) unless otherwise indicated.

^#^A mixed model fitted with period, treatment fixed effect and subjects as the random effect was used in this analysis for Day 1 and Day 10 separately. Log_e_-transformed AUC and C_max_ values were used in the model. The treatment ratios were calculated by back-transforming the difference between the adjusted means.

*Data shown as median (range);

^†^UMEC/VI 62.5/25 μg, n = 11;

^‡^UMEC/VI 62.5/25 μg, n = 8.

AUC_0–2_, area under the concentration—time curve from time zero (pre-dose) to 2 hours; CI, confidence interval; C_max_, maximum observed concentration; N/A = not available (insufficient data to derive measure); t_½_, terminal half-life; t_last_, time of last quantifiable concentration; t_max_, time of occurrence of C_max_; UMEC, umeclidinium; VI, vilanterol.

Following single dosing, VI C_max_ was slightly lower when comparing UMEC/VI 62.5/25 μg with VI monotherapy, and slightly higher for UMEC/VI 125/25 μg compared with VI monotherapy. Conversely, VI AUC_(0–2)_ was higher for UMEC/VI 62.5/25 μg compared with VI monotherapy, and lower for UMEC/VI 125/25 μg compared with VI monotherapy. However, for each comparison of both parameters, the 90% CI for the ratio of adjusted geometric means included 1.0 (Table 3).

Following repeat dosing, VI AUC_0–2_ was lower when comparing UMEC/VI with VI monotherapy (UMEC/VI 62.5/25 μg versus VI monotherapy, 22% lower; UMEC/VI 125/25 μg versus VI monotherapy, 19% lower).

Across all administered doses, VI accumulation was 25–66% based on C_max_ and 17–43% based on AUC_(0–2)_ (S4 Table). Steady-state could not be assessed as all trough concentrations were below the LLOQ.

The inter-subject coefficient of variation following both single- and repeat-dose administration for all the VI PK parameter estimates in the healthy Chinese subjects ranged from 13% to 116% for UMEC/VI 62.5/25 μg and UMEC/VI 125/25 μg, and 20% to 55% for VI 25 μg.

### Safety profile

Twelve subjects experienced ≥1 AE (UMEC/VI 62.5/25 μg, n = 6 [50%]; UMEC/VI 125/25 μg, n = 2 [18%]; UMEC 62.5 μg, n = 2 [17%]; UMEC 125 μg, n = 7 [58%]; VI 25 μg, n = 4 [36%]) (Table 4). All AEs were of mild or moderate intensity and had resolved by the end of the study. No AEs led to withdrawal from the study. No SAEs were reported. Six (30%) subjects reported ≥1 treatment-related AE (UMEC/VI 62.5/25 μg, n = 2 [17%]; UMEC/VI 125/25 μg, n = 2 [18%]; UMEC 62.5 μg, n = 1 [8%]; UMEC 125 μg, n = 4 [33%]; VI 25 μg, n = 1 9%]; Table 5). The most frequently reported treatment-related AE was chest discomfort (total, n = 3 [15%]; UMEC/VI 62.5/25 μg, n = 2 [17%]; UMEC 125 μg, n = 2 [17%]; VI 25 μg, n = 1 [9%]). All AEs resolved before the next treatment sequence started, except two AEs (urine acid increased and blood bilirubin increased) which were considered unrelated to study medication.

**Table 4:** Summary of all adverse events (safety population). *

	UMEC/VI 62.5/25 μg (N = 12)	UMEC/VI 125/25 μg (N = 11)	UMEC 62.5 μg (N = 12)	UMEC125 μg (N = 12)	VI 25 μg (N = 11)
Subject with any adverse event, n (%)	6 (50)	2 (18)	2 (17)	7 (58)	4 (36)
Chest discomfort	2 (17)	0	0	2 (17)	1 (9)
Decreased neutrophil count	2 (17)	0	0	2 (17)	1 (9)
Decreased white blood cell count	3 (25)	0	0	1 (8)	1 (9)
Increased blood bilirubin	0	0	1 (8)	2 (17)	0
Anemia	0	0	0	0	1 (9)
Increased blood uric acid	1 (8)	0	1 (8)	0	0
Constipation	0	1 (9)	0	1 (8)	0
Decreased appetite	1 (8)	0	0	0	0
Dry throat	0	0	0	1 (8)	0
Epistaxis	0	0	0	1 (8)	0
Increased heart rate	0	1 (9)	0	0	0
Influenza like symptoms	0	0	0	1 (8)	0
Nasopharyngitis	0	0	0	0	1 (9)
Palpitations	0	0	0	0	1 (9)
Pericoronitis	1 (8)	0	0	0	0
Pyrexia	0	0	0	0	1 (9)

*n numbers represent the number of subjects experiencing an event, which may be different to the total number of events.

UMEC, umeclidinium; VI, vilanterol.

**Table 5:** Summary of all treatment-related adverse events (safety population). *

	UMEC/VI 62.5/25 μg (N = 12)	UMEC/VI 125/25 μg (N = 11)	UMEC 62.5 μg (N = 12)	UMEC125 μg (N = 12)	VI 25 μg (N = 11)
Subject with any treatment related adverse event, n (%)	2 (17)	2 (18)	1 (8)	4 (33)	1 (9)
Chest discomfort	2 (17)	0	0	2 (17)	1 (9)
Increased blood bilirubin	0	0	1 (8)	1 (8)	0
Constipation	0	1 (9)	0	1 (8)	0
Decreased appetite	1 (8)	0	0	0	0
Dry throat	0	0	0	1 (8)	0
Epistaxis	0	0	0	1 (8)	0
Increased heart rate	0	1 (9)	0	0	0
Palpitations	0	0	0	0	1 (9)

*n numbers represent the number of subjects experiencing an event, which may be different to the total number of events.

UMEC, umeclidinium; VI, vilanterol.

**Fig 4:**
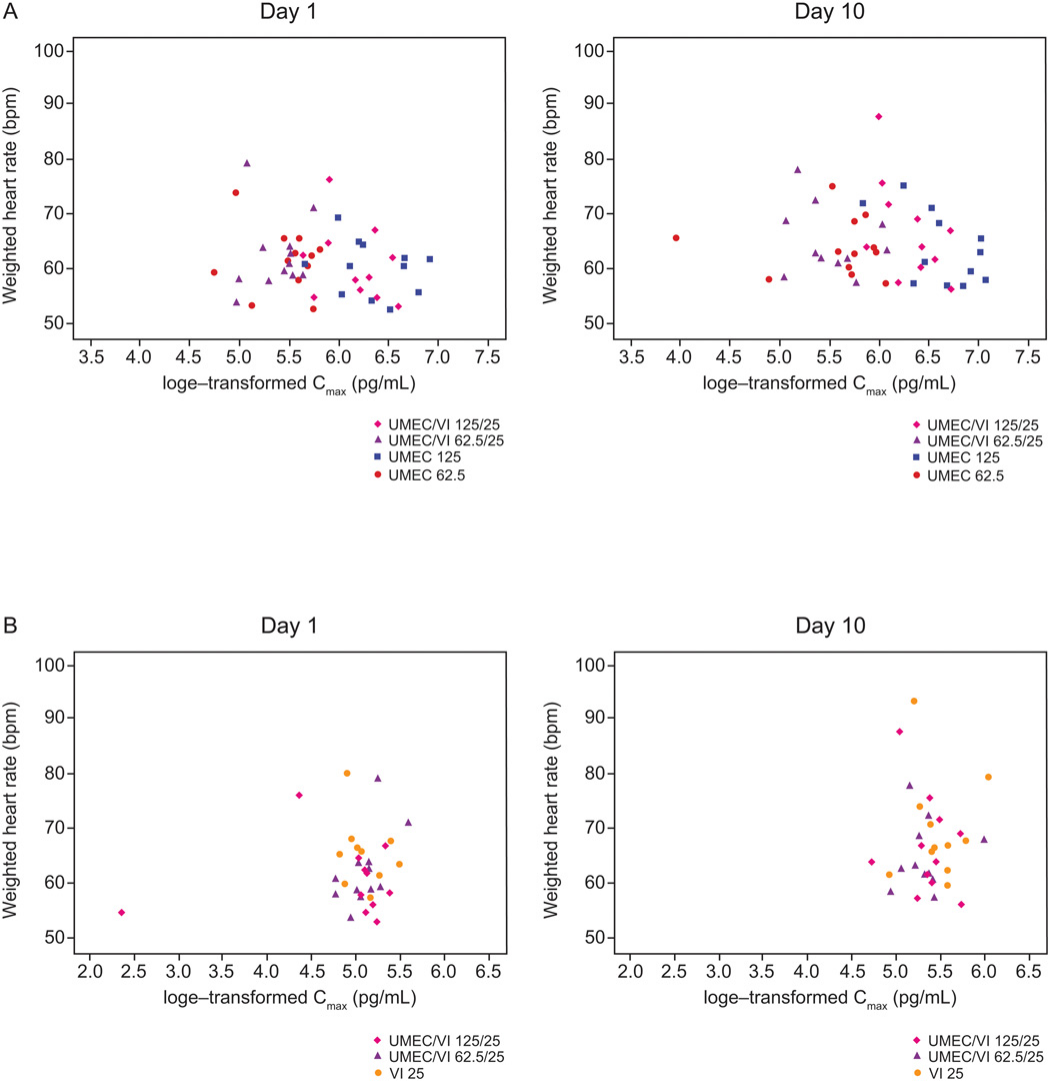
Log_e_-transformed C_max_ against weighted mean heart rate (0–4 h) for UMEC (A) and VI (B). bpm, beats per minute; C_max_, maximum plasma concentration; PK, pharmacokinetic; UMEC, umeclidinium; VI, vilanterol.

**Fig 5:**
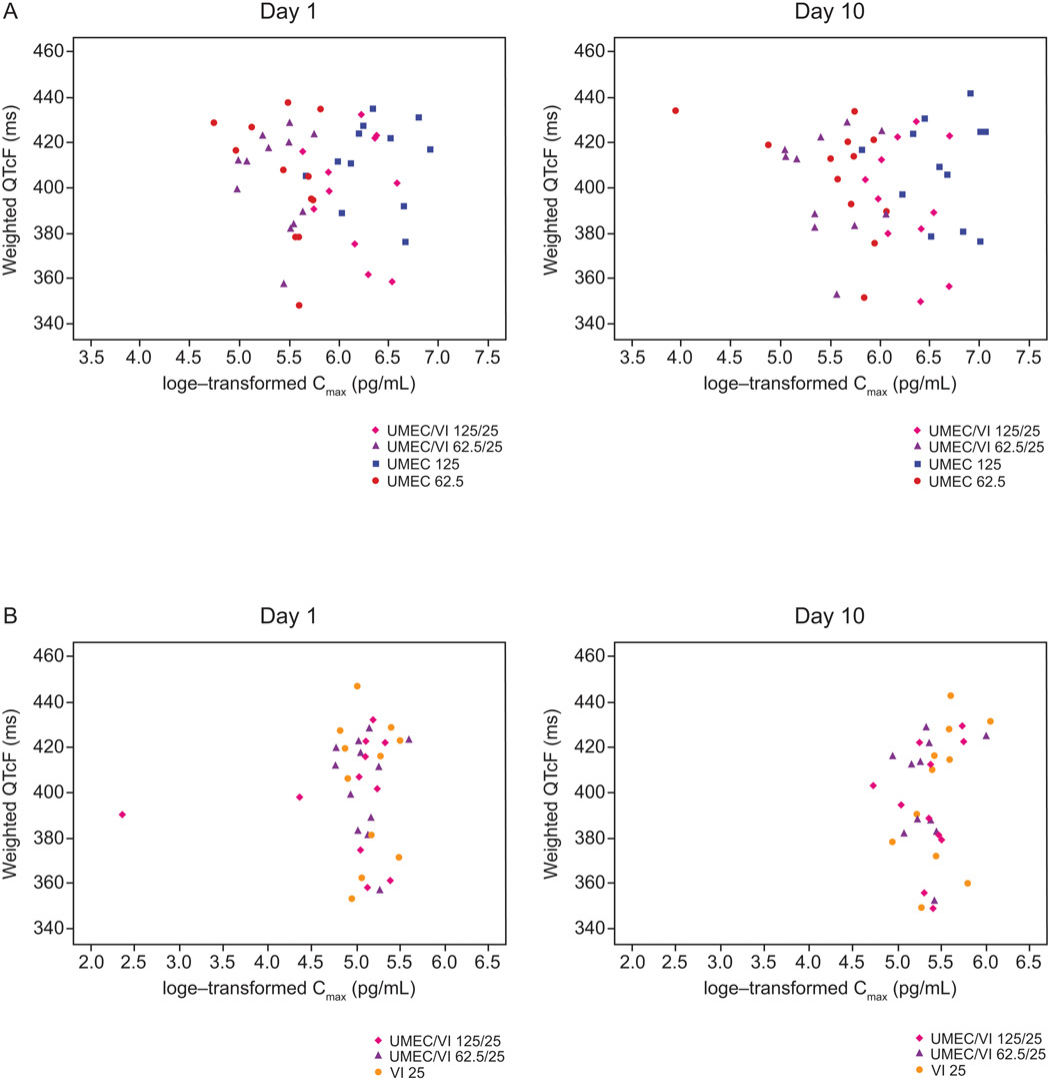
Log-transformed C_max_ against weighted QTcF (0–4 h) for UMEC (A) and VI (B). C_max_, maximum plasma concentration; PK, pharmacokinetic; UMEC, umeclidinium; VI, vilanterol.

No clinically important changes in vital signs, HR, or ECG parameters were observed based on the mean data for each treatment group. No concentration-related effects on 0–4 h weighted mean HR and 0–4 h weighted mean QTcF parameters were observed for UMEC or VI, delivered as combination therapy or as monotherapies (Fig. 4 and Fig. 5). Increased HR was reported in one subject in the UMEC/VI 125/25 μg group. However, the increase did not meet the predefined criteria of clinically relevant importance (<45 beats per minute and ≥110 beats per minute) and resolved without treatment. Overall, no clinically significant changes in laboratory values from baseline were reported during the study for any treatment group; one subject had a high total bilirubin value at screening and over the three treatment periods (UMEC 62.5 μg/UMEC 125 μg/VI 25 μg), which was considered an AE during treatment with UMEC 62.5 μg and VI 25 μg.

## Discussion

This study assessed the PK and tolerability of UMEC/VI combination therapy (62.5/25 μg and 125/25 μg) and their constituent monotherapies following single and repeat inhaled doses in healthy subjects of Chinese heritage.

The results indicated rapid absorption, distribution, and elimination from the systemic circulation of both UMEC and VI. These results are consistent with studies of UMEC monotherapy in predominantly White patients with COPD,[16–18] and with a study of UMEC monotherapy and UMEC/VI combination therapy in healthy Japanese subjects.[19] The results of the present study suggest that systemic exposure was not substantially different for UMEC/VI compared with UMEC or VI as monotherapy following single and repeat dosing, as the 95% CIs overlapped and the 90% CIs for the ratio of the adjusted geometric means for C_max_ and AUC_(0–2)_ included 1.0 in most comparisons. In line with these findings, Goyal et al., 2014 recently reported no apparent PK interactions when UMEC and VI were co-administered in patients with COPD of predominantly White Caucasian/European heritage.[12] Assessments of PK interactions in healthy Japanese subjects were inconclusive, where UMEC C_max_ (but not AUC) was higher following combination therapy compared with UMEC monotherapy. Conversely for VI, AUC (but not C_max_) was higher following combination therapy compared with VI monotherapy.[19] Although factors contributing to higher UMEC C_max_ and VI AUC following combination treatment were unclear, the combination in the Japanese study was administered through concomitant use of two separate inhalers. The study did not specify order of dosing of the two separate inhalers and there was a formulation difference in treatments due to increased magnesium stearate exposure using two separate inhalers for concurrent administration compared with UMEC and VI alone. [19] Regardless of the factors contributing to the increased exposure, the results of another repeat dose, combination study indicated that, when administered simultaneously in a single inhaler device, VI did not have an effect on systemic exposure of UMEC following single or repeat doses. [20]

Overall, the repeat-dose UMEC and VI PK profiles in healthy Chinese subjects observed in this study were generally consistent with the repeat-dose PK profiles observed in healthy Japanese and Western (mostly White) subjects, although there was a trend of higher UMEC exposure (<2-fold) in the East Asian subjects,[19, 21–23] which has not been shown to be clinically significant. Importantly, population PK analyses of UMEC and VI data in 1635–1637 subjects with COPD also indicated that ethnicity is not a significant covariate for UMEC or VI apparent clearance or apparent volume of distribution.[12]

Systemic drug levels achieved with inhaled pharmacotherapies potentially elicit unwanted pharmacological effects. The low observed systemic exposures of UMEC and VI could be considered advantageous to avoid any potential systemic adverse effects. Indeed, the single- and repeat-dose administration of UMEC/VI and UMEC and VI monotherapies was well tolerated, with no clinically meaningful findings in ECG assessments or HR. These findings on safety and tolerability are consistent with previous reports at these doses.[5,6,17,18,24,25]

A limitation of the present study is that despite the high sensitivity of the bioanalytical methods, many plasma concentration measurements of UMEC and VI were below the LLOQ, precluding full characterization of the PK profile of UMEC and VI.

In conclusion, single- and repeat-dose administration of UMEC/VI combination therapy in healthy Chinese subjects did not result in substantial differences in systemic exposure compared with the constituent monotherapies. Systemic UMEC exposure was generally consistent with previous studies in healthy Western, mostly White subjects. UMEC/VI combination therapy and UMEC and VI monotherapies were well tolerated, and no safety concerns were raised.

## Supporting Information

S1 Checklist CONSORT checklist. (PDF)

S1 Protocol Study protocol. (PDF)

S1 Supporting Information Supplementary Methods. (DOC)

S1 Table Treatment sequences for 20 subjects in this balanced, incomplete-block, three-way crossover study. A: UMEC/VI 62.5/25 μg; B: UMEC/VI UMEC/VI 125/25 μg; C: UMEC 62.5 μg; D: UMEC 125 μg; E: VI 25 μg.
(DOCX)

S2 Table Analysis of UMEC PK parameters to determine attainment of steady-state (based on visual observation of individual trough concentrations on Days 6–11*). A mixed model with treatment, day and treatment-by-day interaction as covariates was used in the steady-state evaluation.*Day 11 was the 24-h post-dose concentration on Day 10. CI, confidence interval; PK, pharmacokinetic; UMEC, umeclidinium; VI, vilanterol.
(DOC)

S3 Table Analysis of UMEC PK parameters to assess accumulation: ratio of geometric means (Day 10/Day 1). A mixed model fitted with day as a fixed effect and subject as the random effect was used in the accumulation assessment (by treatment). AUC_(0–2)_, area under the concentration-time curve from time zero to 2 h; CI, confidence interval; C_max_, maximum plasma concentration; PK, pharmacokinetic; UMEC, umeclidinium; VI, vilanterol.
(DOC)

S4 Table Analysis of VI PK parameters to assess accumulation: ratio of geometric means (Day 10/Day 1). A mixed model fitted with day as the fixed effect and subjects as the random effect was used in the accumulation assessment (by treatment). AUC_(0–2)_, area under the concentration-time curve from time zero to 2 h; CI, confidence interval; C_max_, maximum plasma concentration; PK, pharmacokinetic; UMEC, umeclidinium; VI, vilanterol.
(DOC)
