# Supplementary material for: Pharmacokinetics and Tolerability of Inhaled Umeclidinium and Vilanterol Alone and in Combination in Healthy Chinese Subjects: A Randomized, Open-Label, Crossover Trial
Source: PLoS One. 2015 Mar 27;10(3):e0121264. doi: 10.1371/journal.pone.0121264 (PMC4376748; doi:10.1371/journal.pone.0121264)
Supplement: S1 Protocol — (PDF) [file pone.0121264.s002.pdf]

Division: Worldwide Development
Information Type: Protocol Amendment

**Title:**	A Randomized, Open Label, 3-Period Crossover, Balanced Incomplete Block Study To Evaluate The Pharmacokinetics Of Umeclidinium Bromide and Vilanterol Trifenatate as Monotherapies and Concurrently in Healthy Chinese Subjects.

Compound Number: GSK573719+GW642444

Effective Date: 09-APR-2013

Protocol Amendment Number: 01

Description:

Vilanterol trifenatate (VI, GSK compound number GW642444) is a potent and selective long-acting β2 agonist; Umeclidinium bromide (UMEC, GSK compound number GSK573719) is a long-acting, inhaled, muscarinic receptor antagonist (or anticholinergic). Both compounds are in development combination administrated once daily for the treatment of Chronic Obstructive Pulmonary Disease (COPD).

This study is a randomized, open label, three-period crossover, balanced incomplete block study which will assess the pharmacokinetics (PK), safety and tolerability of UMEC (62.5μg and 125μg) and VI (25μg) as monotherapies and combinations in 20 healthy Chinese subjects. Each subject will receive three of five possible treatments for 10 days each.

Blood samples for PK analysis will be taken at designed timepoints. Safety will be assessed by measurement of ECG QTcF, heart rate, blood pressure, and safety laboratory data and review of adverse events.

Subject: [LAMA (long-acting muscarinic acetylcholine receptor antagonist), LABA (long-acting beta adrenergic receptor agonist), healthy Chinese subjects, safety, tolerability, pharmacokinetics, Umeclidinium bromide (UMEC), GSK573719, Vilanterol triflenatate (VI), GW642444.]

Author(s): [redacted] (MDC, China); [redacted] (MDC, China); [redacted] (MDC, China); [redacted] (MDC, China); [redacted] (CPK M&S, US); [redacted] (MDC, US); [redacted] (CPMS, UK); [redacted] (Stat, UK); [redacted] (MDC, China)

Copyright 2013 the GlaxoSmithKline group of companies. All rights reserved. Unauthorised copying or use of this information is prohibited.

Revision Chronology:

2010N111573_00	2012-SEP-17	Original
2010N111573_01	2013-APR-09	Amendment No.: 1 Details correction and procedure clarification

SPONSOR SIGNATORY:

[redacted] ______________________ April 9, 2013
Date

[redacted]

VP, Medicines Development
GSK (China) R&D Co. Ltd

SPONSOR/MEDICAL MONITOR INFORMATION PAGE

Medical Monitor and Sponsor Contact Information:

**Role**	Name	Day Time Phone Number	After-hours Phone/Cell/ Pager Number	Fax Number	GSK Address
Primary Medical Monitor	[redacted]	[redacted]	[redacted]	[redacted]	No.1 Building, 917 Halei Road, Zhangjiang Hi-tech Park, Pudong Shanghai 201203, China
Secondary Medical Monitor	[redacted]	[redacted]	[redacted]	[redacted]	Ocean Center, Block A, 56 Dongsihuan Zhonglu,, Chaoyang District, Beijing , 100025, China
Tertiary Medical Monitor	[redacted]	[redacted]			Stockley Park West, Uxbridge, Middlesex, UB11 1BT United Kingdom
SAE fax number				[redacted]	

Sponsor Registered Address:

China GlaxoSmithKline Research & Development Center
No.1 Building, 917 Halei Road,
Zhangjiang Hi-tech Park, Pudong Shanghai
China. 201203

Regulatory Agency Identifying Number(s): SFDA CTP number 2012L00463

INVESTIGATOR AGREEMENT PAGE

For protocol 2010N111573_01

I confirm agreement to conduct the study in compliance with the protocol, as amended by this protocol amendment.

I acknowledge that I am responsible for overall study conduct. I agree to personally conduct or supervise the described clinical study.

I agree to ensure that all associates, colleagues and employees assisting in the conduct of the study are informed about their obligations. Mechanisms are in place to ensure that site staff receives the appropriate information throughout the study.

Investigator Name: ______________________________

______________________________ ____________________

Investigator Signature Date

TABLE OF CONTENTS

PAGE

ABBREVIATIONS 9

1. INTRODUCTION 11
   1.1. Background 11
      1.1.1. COPD and clinical management of the disease 11
      1.1.2. Umeclidinium bromide (UMEC) 11
      1.1.3. Vilanterol trifenatate (VI) 12
      1.1.4. UMEC and VI concurrent dosing 12
   1.2. Rationale 13
      1.2.1. Study Rationale 13
      1.2.2. Dose Rationale 13
   1.3. Summary of Risk Management 14

2. OBJECTIVE(S) 14
   2.1. Primary 14
   2.2. Secondary 14

3. ENDPOINT(S) 15
   3.1. Primary 15
   3.2. Secondary 15

4. INVESTIGATIONAL PLAN 15
   4.1. Study Design/Schematic 15
   4.2. Discussion of Design 16
   4.3. Treatment Assignment 16
   4.4. Investigational Product and Other Study Treatment Dosage/Administration 17
   4.5. Stopping Criteria 18
      4.5.1. Liver Chemistry Stopping Criteria 19
      4.5.2. QTcF Withdrawal Criteria 19
      4.5.3. Other Dose Adjustment/Stopping Safety Criteria 19
   4.6. Time and Events Table 20

5. STUDY POPULATION 23
   5.1. Number of Subjects 23
   5.2. Eligibility Criteria 23
      5.2.1. Inclusion Criteria 23
      5.2.2. Exclusion Criteria 24
      5.2.3. Other Eligibility Criteria Considerations 25

6. DATA ANALYSIS AND STATISTICAL CONSIDERATIONS 26
   6.1. Hypotheses and Treatment Comparisons 26
   6.2. Sample Size Considerations 26
      6.2.1. Sample Size Assumptions 26
      6.2.2. Sample Size Sensitivity 26
      6.2.3. Sample Size Re-estimation 27
   6.3. Data Analysis Considerations 27
      6.3.1. Interim Analysis 27
      6.3.2. Final Analyses 27

6.3.2.1. Analysis Population 27
6.3.2.2. Pharmacokinetic Analyses....27
6.3.2.2.1. Raw Plasma Concentrations....27
6.3.2.2.2. Derived Plasma Pharmacokinetic Parameters....27
6.3.2.2.3. Steady State....28
6.3.2.2.4. Assessment of accumulation 28
6.3.2.2.5. Time-Invariance....29
6.3.2.2.6. Treatment Comparison of Systemic Exposure....29
6.3.2.3. Pharmacokinetic/Pharmacodynamic Analyses 29
6.3.2.4. Safety Analyses....29

7. STUDY ASSESSMENTS AND PROCEDURES 29
7.1. Demographic/Medical History Assessments 30
7.2. Safety 30
7.3. Pregnancy 32
7.3.1. Time period for collecting pregnancy information 32
7.3.2. Action to be taken if pregnancy occurs 32
7.4. Pharmacokinetics 33
7.4.1. Blood Sample Collection....33
7.4.2. Sample Analysis 33

8. LIFESTYLE AND/OR DIETARY RESTRICTIONS....33
8.1. Contraception Requirements 33
8.1.1. Female Subjects....33
8.1.2. Male Subjects....34
8.2. Meals and Dietary Restrictions 35
8.3. Caffeine, Alcohol, and Tobacco 35
8.4. Activity....35

9. CONCOMITANT MEDICATIONS AND NON-DRUG THERAPIES 35
9.1. Permitted Medications 35
9.2. Prohibited Medications....36
9.3. Non-Drug Therapies 36

10. COMPLETION OR EARLY WITHDRAWAL OF SUBJECTS 36
10.1. Subject Completion....36
10.2. Subject Withdrawal Criteria....36
10.3. Subject Withdrawal Procedures....36
10.3.1. Subject Withdrawal from Study....36
10.4. Treatment after the End of the Study 37
10.5. Screen and Baseline Failures 37

11. MADE AVAILABLE TO THE MONITOR FOR MONITORING STUDY TREATMENT 37
11.1. Blinding....37
11.2. Packaging and Labeling....37
11.3. Preparation/Handling/Storage/Accountability....37
11.4. Assessment of Compliance 38
11.5. Treatment of Study Treatment Overdose....38

12. ADVERSE EVENTS (AE) AND SERIOUS ADVERSE EVENTS (SAE) 38
12.1. Definition of Adverse Events 39
12.2. Definition of Serious Adverse Events 40
12.3. Method of Detecting AEs and SAEs 41
12.4. Recording of AEs and SAEs 41
12.5. Evaluating AEs and SAEs 42
12.5.1. Assessment of Intensity 42
12.5.2. Assessment of Causality 42
12.6. Follow-up of AEs and SAEs 43
12.7. Prompt Reporting of SAEs to GSK 43
12.8. Regulatory Reporting Requirements for SAEs 43

13. LIVER CHEMISTRY FOLLOW-UP PROCEDURES 44

14. STUDY CONDUCT CONSIDERATIONS 46
14.1. Posting of Information on Clinicaltrials.gov 46
14.2. Regulatory and Ethical Considerations, Including the Informed Consent Process 46
14.2.1. Urgent Safety Measures 47
14.3. Quality Control (Study Monitoring) 47
14.4. Quality Assurance 47
14.5. Study and Site Closure 47
14.6. Records Retention 48
14.7. Provision of Study Results to Investigators, Posting to the Clinical Trials Register and Publication 48
14.8. Data Management 49

15. REFERENCES 50

16. APPENDICES 51
16.1. Appendix 1: Liver Safety Algorithms 51
16.2. Appendix 2: Amendment 1 52

ABBREVIATIONS

AE	Adverse Event
ALT(SGPT)	Alanine aminotransferase (glutamate pyruvate transaminase)
AST(SGOT)	Aspartate aminotransferase (glutamate oxaloacetate transaminase
AUC	Area under concentration-time curve
$AUC_{0-\infty}$	Area under the concentration-time curve from time zero (pre-dose) extrapolated to infinite time
AUC_{0-t}	Area under the concentration-time curve from time zero (pre-dose) to last time of quantifiable concentration
$AUC_{0-t'}$	AUC from zero to the last common quantifiable time point in the dose group
$AUC_{0-\tau}$	Area under the concentration-time curve over the dosing interval
BP	Blood pressure
BUN	Blood urea nitrogen
CI	Confidence Interval
C_{max}	Maximum observed concentration
COPD	Chronic Obstructive Pulmonary Disease
CL/F	Systemic clearance of parent drug
CPK	Creatine phosphokinase
CRF	Case Report Form
CV	Coefficient of variance
DF	Degree of Fluctuation of C_{min-ss} and C_{max-ss}
DPI	Dry Powder Inhaler
DMPK	Drug Metabolism and Pharmacokinetics
ECG	Electrocardiogram
FDA	Food and Drug Administration
FEV_1	forced expiratory volume in 1 second
FSH	Follicle Stimulating Hormone
GCP	Good Clinical Practice
GGT	Gamma glutamyltransferase
GSK	GlaxoSmithKline
HBsAg	Hepatitis B surface antigen
HIV	Human Immunodeficiency Virus
HR	Heart rate
IB	Investigator's Brochure
ICH	International Conference on Harmonization of Technical Requirements for Registration of Pharmaceuticals for Human Use
IEC	Independent Ethics Committee
IP	Investigational Product
IRB	Institutional Review Board
IU	International Unit
LABA	long-acting beta agonist
LAMA	long-acting muscarinic acetylcholine receptor agonist
LLQ	Lower limit of quantification
MCH	Mean corpuscular hemoglobin

MCHC	Mean corpuscular hemoglobin concentration
MCV	Mean corpuscular volume
MSDS	Material Safety Data Sheet
NDPI	Novel dry powder inhaler
NQ	Non-quantifiable concentration measured as below LLQ
PD	Pharmacodynamic
PK	Pharmacokinetic
QTcF	QT duration corrected for heart rate by Fridericia's formula
RAP	Reporting and Analysis Plan
RBC	Red blood cells
RC_{max}	$RC_{max} = C_{max}$ (day 10) /C_{max} (day 1)
Ro	Observed accumulation ratio calculated as $AUC_{0-\tau}$ (day10)/ $AUC_{0-\tau}$ (day1)
SAE	Serious adverse event(s)
SD	Standard deviation
SOP	Standard Operating Procedure
SPM	Study Procedures Manual
$t_{½}$	Terminal phase half-life
τ	Dosing interval
t_{last}	Time of last quantifiable concentration
t_{max}	Time of occurrence of Cmax
µg	Microgram
ULN	Upper limit of normal
UK	United Kingdom
US	United States
Vd/F	Volume of Distribution
WBC	White blood cells

Trademark Information

Trademarks of the GlaxoSmithKline group of companies
NONE

Trademarks not owned by the GlaxoSmithKline group of companies
InForm
WinNonlin

1. INTRODUCTION

1.1. Background

1.1.1. COPD and clinical management of the disease

COPD is a preventable and treatable disease which is characterized by airflow limitation that is not fully reversible. This airflow limitation is progressive and is associated with an abnormal inflammatory response of the lung to noxious particles or gases ([Global Initiative for Chronic Obstructive Lung Disease guidelines [GOLD, 2011]). Pharmacological management of COPD attempts to reduce symptoms, improve quality of life, optimize lung function, reduce exacerbations and improve exercise tolerance.

Inhaled bronchodilators, including β_2-adrenoreceptor agonists and anticholinergics are the mainstays of therapy in patients diagnosed with COPD. Bronchodilator classes include drugs with a short duration of action (e.g. the β_2 agonist salbutamol, and the anticholinergic ipratropium bromide) or long duration of action (e.g. the β_2 agonist salmeterol, and the anticholinergic tiotropium bromide). The choice of therapeutic agent depends largely on individual response in terms of symptom relief and adverse effects, as all are effective at improving lung function as measured by FEV_1 (Forced Expiratory Volume in 1 Second).

Inhaled short acting, β_2 agonists have been the mainstays of therapy in patients diagnosed with COPD and have been proven to be effective and generally well tolerated. Stimulation of the β_2 adrenoreceptor in the lung relaxes bronchial smooth muscle cells which results in bronchodilation. Unwanted systemic side effects related to β_2 agonist treatment such as tachycardia, tremor, hyperglycaemia and hypokalaemia are generally mild and are limited by local administration and also tend to show tachyphylaxis.

Anticholinergic bronchodilators function by blocking endogenous cholinergic tone. Ipratropium bromide and oxitropium bromide are non-selective antagonists which act at the M1, M2 and M3 receptors, and consequently may have bronchodilating activity through the M1/M3 receptors, and bronchoconstricting activity through the M2 receptor. Tiotropium bromide is a more recent quaternary ammonium anticholinergic that shows kinetic selectivity for the M1 and M3 receptor subtypes over the M2 subtype. All three of the above drugs are poorly absorbed, which limit the troublesome systemic effects observed with belladonna alkaloids.

1.1.2. Umeclidinium bromide (UMEC)

UMEC (GSK573719) is a quinuclidine derivative which is a potent pan-active mAChR antagonist (anticholinergic) and is being developed for once daily treatment of COPD. It is a high affinity specific reversible mAChR antagonist that has rapid on and slow-off kinetics at the human M3 muscarinic receptor subtype. It has been shown to be an effective long acting bronchodilator in humans by inhalation.

Detailed information relating to non-clinical pharmacology, safety pharmacology, PK and metabolism, toxicology and other pre-clinical data can be found in the Umeclidinium (GSK573719) Investigator's Brochure (IB) [GlaxoSmithKline Document Number RM2006/00835/08].

1.1.3. Vilanterol trifenatate (VI)

VI (GW642444) is a novel, potent and selective long acting inhaled β_2 adrenoreceptor agonist (LABA), which is being developed for once-daily treatment of asthma and COPD.

Please refer to the Vilanterol (GW642444) Investigator's Brochure [GlaxoSmithKline Document Number SM2003/00028/09] for further details on the clinical studies conducted and completed to date.

1.1.4. UMEC and VI concurrent dosing

UMEC/VI combination was evaluated in healthy subjects (DB2113208 and DB2113590) and COPD patients (DB2113120).

In study DB2113208, where individual components of the combination were administered using separate inhalers, pharmacokinetic analyses of area under the curve (AUC) showed no clear evidence of a difference in UMEC systemic exposure when delivered concurrently with VI Inhalation Powder compared with UMEC Inhalation Powder administered alone. The ratio of adjusted mean for C_{max} showed evidence of a 30% increase in exposure [95% CI: 4% to 64% increase]) in the concurrent treatment arm compared with UMEC Inhalation Powder alone. For VI, C_{max} analysis showed no clear evidence of a difference in VI systemic exposure when delivered concurrently with UMEC Inhalation Powder compared with VI Inhalation Powder administered alone. The ratio of adjusted mean for AUC showed evidence of an on average 39% increase [95% CI: 7% to 80% increase]) when concurrent treatment was administered compared with VI alone.

Unlike DB2113208, in Study DB2113950, which used NDPI similar to phase III formulations, UMEC steady-state systemic exposure in terms of AUC and C_{max} data both on Days 8 and 13 showed no increase in UMEC systemic exposure in the presence of VI and systemic exposure was somewhat lower in the UMEC/VI Inhalation Powder arm compared with the UMEC Inhalation Powder arm. These data indicate that VI did not have an effect on systemic exposure of UMEC, thereby supporting the lack of a pharmacokinetic drug-drug interaction between UMEC and VI.

The pharmacokinetic and safety profiles of UMEC/VI in monotherapies (AC4113377, DB1112146, and DB1112017) or combinations (DB2113208) in healthy Japanese subjects have been assessed. This study in healthy Chinese subjects is expected to get similar results.

The detailed information related to nonclinical/clinical pharmacology, pharmacokinetics and toxicology can be found in GSK573719+GW642444 Investigator's Brochure (IB) [GlaxoSmithKline Document Number RM2009/00437/02]

1.2. Rationale

1.2.1. Study Rationale

Both UMEC and VI have the potential for once daily administration and the ability to combine them in the novel dry powder inhaler as a dual product means that these two molecules demonstrate promise as a combination treatment. Both UMEC and VI have been administered to healthy subjects and patients overseas as a dry powder containing lactose and magnesium stearate.

LABA and LAMA therapies are co-prescribed for COPD in clinical practice and an additional therapeutic benefit of concurrent therapy has been demonstrated. Study DB2113120 in COPD patients demonstrated an additional effect on FEV_1 in a cross-study comparison when UMEC and VI were administrated as a combination treatment via a single Novel Dry Powder Inhaler (Novel DPI).

This study will assess pharmacokinetics, safety and tolerability of this combination for the first time in healthy Chinese volunteers and will facilitate the participation of Chinese subjects to assess if the results are in compliance with the results obtained overseas.

1.2.2. Dose Rationale

The safety, tolerability, pharmacokinetics and pharmacodynamics of UMEC have been evaluated in clinical studies in healthy subjects and COPD patients following single and repeat dose administration in overseas subjects. These clinical studies have shown UMEC to be well-tolerated when single and repeat inhaled doses of UMEC of up to 1000µg were administered. The 62.5µg and 125µg UMEC repeat doses were studied in COPD patients in studies AC4113073 (14 days), AC4115321 (7 days) and AC4115408 (3 months).In healthy subjects (AC4105209, AC106889, AC4110106, AC4113377) UMEC was rapidly absorbed with median t_{max} values of 5 minutes after single inhaled dosing and after repeat inhaled dosing, and showed somewhat more than dose proportional increase over the range in these studies. The 62.5µg and 125µg UMEC doses in this study are currently being used in Phase III studies globally.

The 25µg VI dose in this study is the dose being used in phase III studies globally. The VI formulation with magnesium stearate to be administered in this study was previously administered in a 14-day repeat dose study in healthy subjects (VI doses 25, 50 and 100µg; study B2C108784) and as a single dose in asthmatic patients (VI doses 25, 50 and 100µg; study B2C106996). In B2C108784, VI was well tolerated with an adverse event incidence similar to that seen with placebo. In the single dose study in patients with persistent asthma (study B2C106996) VI (25, 50 and 100µg) was well tolerated producing only small but statistically significant pharmacodynamic effects on physiological parameters typically at higher doses of long-acting beta-agonists. These were most frequently seen after administration of 100µg VI and were considered of no clinical significance.

1.3. Summary of Risk Management

- The study is being run at a clinical pharmacology contract research organization approved by SFDA, which has relevant emergency services required officially, and has access to the hospital emergency room and the hospital intensive care unit.
- Subjects will be under medical supervision for each investigational product administration while vital signs are assessed. Subjects will remain under medical supervision if the Investigator has found any abnormal safety data.
- Subjects will be monitored during the post dose period for known effects of muscarinic antagonists and in particular tachycardia and arrhythmia. Acceptable limits for changes in these parameters have been established and safety criteria for consideration of subject withdrawal are included in the protocol, see Section 4.5.
- Subjects will be monitored during the post dose period for known effects of β_2 agonists and in particular tachycardia. Acceptable limits for changes in these parameters have been established and safety criteria for consideration of subject withdrawal are included in the protocol, see Section 4.5.

2. OBJECTIVE(S)

2.1. Primary

- To evaluate the pharmacokinetics of UMEC following single and once daily 10-day repeat inhaled doses at 62.5µg and 125µg in Chinese healthy subjects when administrated alone.
- To evaluate the pharmacokinetics of UMEC following single and once daily 10-day repeat inhaled doses at 62.5µg and 125µg in Chinese healthy subjects when in combination with VI (25µg).
- To evaluate the pharmacokinetics of VI following single and once daily 10-day repeat inhaled doses at 25µg in Chinese healthy subjects when administrated alone.
- To evaluate the pharmacokinetics of VI following single and once daily 10-day repeat inhaled doses at 25µg in Chinese healthy subjects when in combination with UMEC (62.5µg and 125µg).

2.2. Secondary

- General safety and tolerability endpoints: adverse events, heart rate, systolic and diastolic blood pressure, 12- lead ECG (QTcF), and clinical laboratory safety tests.

3. ENDPOINT(S)

3.1. Primary

- Plasma concentrations and derived pharmacokinetic parameters of UMEC and VI
  - Single dose: C_{max}, t_{max}, t_{last}, AUC_{0-t}, and $t_{1/2}$, CL/F, Vd/F, $AUC_{0-\infty}$, and $AUC_{0-t'}$, as data permitted.
  - Repeat dose:C_{max}, C_{τ}, t_{max}, t_{last}, AUC_{0-t}, $AUC_{0-\tau}$, $t_{1/2}$, CL/F, Ro and RC_{max}, $AUC_{0-t'}$, DF, as data permitted

3.2. Secondary

- Safety and tolerability
  - Vital signs: blood pressure (systolic and diastolic) and heart rate
  - 12-lead ECG (including weighted mean and maximum QTcF 0-4h on Day 1 and Day 10)
  - Laboratory tests (clinical chemistry, haematology and urinalysis)
  - Adverse events

4. INVESTIGATIONAL PLAN

4.1. Study Design/Schematic

This is a single centre, randomized, open label, 3-period crossover, balanced incomplete block study in healthy Chinese subjects.

All subjects will attend the unit for screening within 21 days of their first of three dosing periods. Each subject will receive three of the five treatment regimens described in Table 1 below.

Table 1 Regimens of the study

Regimens	Treatments (inhaled once daily for 10 days)	
	NDPI Strip 1	NDPI Strip 2
A	UMEC 62.5µg	VI 25µg
B	UMEC 125µg	VI 25µg
C	UMEC 62.5µg	N/A
D	UMEC 125µg	N/A
E	VI 25µg	N/A

N/A=Not Applicable

The order in which these treatments are administered will be in accordance with the randomisation schedule, and there will be a minimum washout period of 7-14 days between periods.

Each subject will be admitted to the unit in the evening prior to Day 1 and remain resident until all assessments completed on D12. All subjects will be scheduled for a follow up visit, 7-10 days after their final dose.

The overall maximum duration (screening to follow-up) for participating subjects will be about 13 weeks.

Protocol waivers or exemptions are not allowed. Therefore, adherence to the study design requirements, including those specified in the Time and Events Table, are essential

4.2. Discussion of Design

This study will evaluate the pharmacokinetics of UMEC (62.5µg and 125µg) and VI (25µg) for the first time administered as single and repeat inhaled doses and in combinations of UMEC/VI (62.5µg/25µg) and UMEC/VI (125µg/25µg) in healthy Chinese subjects. DB2113208, a study in healthy Japanese with similar design using the same drug GSK573219 and VI has been recently completed. A somewhat higher systemic exposure (on average a 30% increase) was observed in the combination arm than the mono therapy arms, however, DB2113950, a similar study in UK, showed no clear difference between of UMEC/VI and the individual UMEC and VI arms. Therefore a pharmacokinetic study of the combination UMEC + VI compared with UMEC and VI administered individually will be conducted in healthy Chinese adult volunteers. The crossover study design will be adopted to reduce the inter-subject variability. The balanced incomplete block design optimally balances a thorough investigation of the 5 treatment regimens and the overall length of study duration for each subject.

4.3. Treatment Assignment

Subjects will be assigned to three-period incomplete cross-over treatment sequence in accordance with the randomization schedule generated by GlaxoSmithKline Biometrics, GlaxoSmithKline R&D, prior to the start of the study, using validated internal software. A replacement schedule will also be set up in place along the randomization schedule in case of a decision made to replace an early withdrew subject.

Prior to the start of the study, 20 subjects will be randomly assigned to one of 20 possible treatment sequences with the aim to achieve 10-12 evaluable subjects for each regimen. A description of each regimen is provided in Table 1. The 20 sequences are based on a balanced incomplete block design. In this design as described below, both treatment and possible pair of treatments occur across periods evenly:

Sequence	Period 1	Period 2	Period 3
1	A	E	D
2	B	A	E
3	C	B	A
4	D	C	B
5	E	D	C
6	A	B	C
7	B	C	D
8	C	D	E
9	D	E	A
10	E	A	B
11	A	D	B
12	B	E	C
13	C	A	D
14	D	B	E
15	E	C	A
16	A	C	E
17	B	D	A
18	C	E	B
19	D	A	C
20	E	B	D

4.4. Investigational Product and Other Study Treatment Dosage/Administration

UMEC/VI Inhalation Powder in the Novel Dry Powder Inhaler contains two blister strips. One strip contains a blend of micronised GSK573719A, lactose monohydrate and magnesium stearate. The second strip contains a blend of micronised GW642444M, lactose monohydrate and magnesium stearate. The Novel Dry Powder Inhaler will deliver, when actuated, the contents of a single blister simultaneously from each of the two blister strips. Each blister strip is a double foil laminate containing 30 blisters per strip.

For individual UMEC treatment, the UMEC blister strip may contain approximately 62.5 or 125µg per blister of GSK573719, as the bromide salt.

For individual VI treatment, the VI blister strip will contain approximately 25µg per blister of GW642444 as the triphenylacetic acid salt. The product is overwrapped, with a dessicant, in a foil laminate.

		Investigational Product		
**Product name:**		UMEC	VI	UMEC and VI[3]
**Formulation description:**	1st strip	GSK573719: micronised drug blended with lactose monohydrate and magnesium stearate[1]	GW642444 micronised drug blended with lactose monohydrate and magnesium stearate[2]	GSK573719: micronised drug blended with lactose monohydrate and magnesium stearate[1]
	2nd strip	N/A	N/A	GW642444: micronised drug blended with lactose monohydrate and magnesium stearate[2]
**Dosage form:**		Novel Dry Powder Inhaler	Novel Dry Powder Inhale	Novel Dry Powder Inhale
**Unit dose strength(s)/Dosage level(s):**		GSK573719 62.5μg or 125μg per blister	GW642444 25μg per blister	GSK573719 62.5μg or 125μg per blister,<br>GW642444 25μg per blister
**Route/ Administration/ Duration:**		Inhaled, once daily single dose for 10 days	Inhaled, once daily single dose for 10 days	Inhaled, once daily single dose for 10 days
**Dosing instructions:**		62.5μg or 125μg once-daily for 10 days	25μg once-daily for 10 days	GSK573719 / GW642444 62.5μg/25μg or 125μg/25μg once-daily for 10 days
**Physical description:**		Dry off white powder	Dry off white powder	Dry off white powder
**Device:**		Novel Dry Powder Inhaler 30 doses per unit	Novel Dry Powder Inhaler 30 doses per unit	GSK573719:Novel Dry Powder Inhaler 30 doses per unit;<br>GW642444: Novel Dry Powder Inhaler 30 doses per unit
**Manufacturer/ source of procurement:**		GSK	GSK	GSK

N/A=Not Applicable

1. Magnesium stearate 0.6% w/w of total drug product
2. Magnesium stearate 1 % w/w of total drug product
3. The combination product (GSK573719 and GW642444) will be administered to the subject by using novel dry powder inhaler devices(NDPI)

4.5. Stopping Criteria

A subject will be withdrawn from the study if they meet any of the stopping criteria in this section.

4.5.1. Liver Chemistry Stopping Criteria

Liver chemistry threshold stopping criteria have been designed to assure subject safety and to evaluate liver event etiology during administration of investigational product and the follow-up period. Investigational product will be stopped if the following liver chemistry stopping criterion is met:

- ALT ≥ 3xULN

Refer to Section 13, Liver Chemistry Follow-up Procedures, for details of the required assessments if a subject meets the above criteria.

4.5.2. QTcF Withdrawal Criteria

A subject that meets the criteria below will be withdrawn from the study. The QT correction formula used to determine discontinuation should be the same one used throughout the study.

- QTcF > 500 msec (machine or manual overread)

Withdrawal decisions are to be based on an average QTcF value of triplicate ECGs. If an ECG demonstrates a prolonged QT interval, obtain 2 more ECGs over a brief period, and then use the averaged QTcF values of the 3 ECGs to determine whether the subject should be discontinued from the study.

4.5.3. Other Dose Adjustment/Stopping Safety Criteria

A subject will be withdrawn from the study if they meet the following stopping criteria:

- Subjects experience intolerable adverse events related to study drug.
- Subjects demonstrate clinically significant and relevant ECG changes.
- Subjects demonstrate clinically significant and relevant changes in laboratory parameters
- Subjects experience resting pulse increases of 40 bpm above the baseline resting heart rate or to a maximum of 130bpm at 2 successive measurements, a minimum of 5 minutes apart and if in the investigator's opinion, following a review of the subject's safety data and discussion with the GSK medical monitor, there are safety concerns.

4.6. Time and Events Table

Detailed procedure and time window please reference to SPM

Table 2 Study Procedures

**Procedure**	Screening	day in each period													Follow-Up
		Day -1	Day 1	Day 2	Day 3	Day 4	Day 5	Day 6	Day 7	Day 8	Day 9	Day 10	Day 11	Day 12	7-10 days after last dose
Admission to the unit		X[1]													
Discharge from the unit														X[1]	
Training session		X													
Informed consent	X														
Medical history, height & weight	X														
Chest Radiography		X[2]													
Physical examination,	X[3]														X[4]
Serology (HIV, Hep B & C)	X														
Drugs of abuse screen	X														
Alcohol breath test and urine cotinine test	X	X													
Laboratory safety tests[5]	X	X[6]											X		X[7]
Vital signs (HR and BP)	X[8]	X	X	X	X	X	X	X	X	X	X	X	X	X	X
12-lead ECG	X[8]		X									X	X	X	X
Urine pregnancy test	X	X[6]											X[9]		
Review entry criteria	X	X													
Randomization		X[10]													
Dosing (AM)			X	X	X	X	X	X	X	X	X	X			

**Procedure**	Screening	day in each period													Follow-Up
		Day -1	Day 1	Day 2	Day 3	Day 4	Day 5	Day 6	Day 7	Day 8	Day 9	Day 10	Day 11	Day 12	7-10 days after last dose
Blood PK sampling			X					X[11]	X[11]	X[11]	X[11]	X	X	X	
Unit visit	X														X
SAE/AE/concurrent medication questioning[12]	X														

1. On day -1 subjects will attend the unit and will be resident until all assessments completed on day12.
2. If a chest X-ray or CT scan is not available within 6 months prior to screening.
3. Physical examination means complete one.
4. Brief physical examination only.
5. Clinical chemistry, haematology & urinalysis.
6. These assessments are taken Day -1 in treatment period 2 and 3.
7. Abnormal lab safety findings post-dosing should be followed up until the tests are normal, stable, no causal relationship with investigational product judged by investigators, or lost contact.
8. Three measurements are to be taken.
9. The test will be taken on Day 11 in the last treatment period.
10. Only on Day-1 in the first treatment period.
11. Blood PK sample will be collected only pre-dosing.
12. AEs and SAEs will be collected from the start of dosing with Investigational Product and until the follow-up visit. However, any SAEs related to study participation or a GSK concomitant medication will be recorded from the time a subject consents to participate in the study and until the follow-up visit.

Table 3 Detailed Time and Events Table for Day 1

Procedure		Day1											
	Pre-dose	0 min	5 min	15 min	30 min	45 min	1h	1.5h	2h	4h	6h	8h	12h
Dosing		X											
Vital Signs (BP and HR)	X		X	X		X		X		X		X	
12-lead ECG	X			X		X		X		X		X	
Blood PK sampling	X		X	X	X		X		X	X	X	X	
SAE/AE/concurrent medication questioning	X												

Table 4 Detailed Time and Events Table for Day 10

Procedure		Day 10												Day 11	Day 12
	Pre-dose	0 min	5 min	15 min	30 min	45 min	1h	1.5h	2h	4h	6h	8h	12h	24h	48h
Dosing		X													
Vital Signs (BP and HR)	X		X	X		X		X		X		X	X	X	X
12-lead ECG	X			X		X		X		X		X	X	X	X
Blood PK sampling	X		X	X	X		X		X	X	X	X	X	X	X
Lab safety tests														X[1]	
SAE/AE/concurrent medication questioning	X														

1. Blood for lab tests will be taken after the blood PK sampling

5. STUDY POPULATION

5.1. Number of Subjects

20 subjects will be randomized to receive the treatments in the assigned sequences. In case the number of evaluable subjects, who complete all dosing and critical assessments, in any treatment group is less than 10 due to early withdrawal, the decision to replace will be made at the discretion of the Sponsor and Investigator so as to ensure at least 10 evaluable subjects per treatment group.

5.2. Eligibility Criteria

5.2.1. Inclusion Criteria

Deviations from inclusion criteria are not allowed because they can potentially jeopardize the scientific integrity of the study, regulatory acceptability or subject safety. Therefore, adherence to the criteria as specified in the protocol is essential.

A subject will be eligible for inclusion in this study only if all of the following criteria apply:

1. Healthy male or females aged 18 - 45 years, inclusive, and the ratio of male and female is 1:1. Healthy as determined by a responsible and experienced physician, based on a medical evaluation including medical history, physical examination, laboratory tests and cardiac monitoring.
2. Body weight ≥ 50kg and body mass index (weight/height^2) within the range of 19 - 24 kg/m^2, inclusive.
3. Male or female subjects at the time of signing the informed consent：

- Female subject who is child-bearing potential should agree to use one of the contraception methods listed in Section 8.1.1. (contraceptives intrauterine device, implantable progesterone device or oral contraceptive) for an appropriate period of time (as determined by the product label or investigator) prior to the start of dosing to sufficiently minimize the risk of pregnancy at that point. The subjects must agree to use contraception until completion of the follow-up visit.
- Male subjects have to agree to use one of the contraception methods listed in Section 8.1.2. This criterion is to be followed from the time of the first dose of study medication until completion of the follow-up visit

4. Normal systolic (90-139mmHg) and diastolic (60-89mmHg) blood pressure at pre-study screening.
5. Subjects who are current non-smokers, who have not used any tobacco products in the 6 month period preceding the screening visit, and have a pack history of ≤10 pack years. (pack years = (cigarettes per day smoked/20) × number of years smoked)).

6. No significant abnormality on 12-lead ECG at screening, QTcF interval must be <450msec (QTcF; machine or manual reading).
7. AST (SGOT), ALT (SGPT), and total-bilirubin ≤ 1.5xULN at screening. No significant clinical abnormality on other laboratory tests.
8. Capable of giving written informed consent, which includes compliance with the requirements and restrictions listed in the consent form.
9. Subjects who are able to use the inhalation device satisfactorily

5.2.2. Exclusion Criteria

Deviations from exclusion criteria are not allowed because they can potentially jeopardize the scientific integrity of the study, regulatory acceptability or subject safety. Therefore, adherence to the criteria as specified in the protocol is essential.

A subject will not be eligible for inclusion in this study if any of the following criteria apply:

1. As a result of medical interview, physical examination or screening investigations, the principle investigator or delegate physician deems the subject unsuitable for the study.
2. History of mental, cardiac, renal, hepatic, significant gastrointestinal or respiratory disease as judged by the investigator
3. A history of breathing problems (i.e. history of asthmatic symptomatology, unless asthma in childhood that has now resolved and no longer requires maintenance therapy which should not be an exclusion).
4. A chest X-ray or computed tomography (CT) scan that reveals evidence of clinically significant abnormalities. A chest X-ray must be taken at day -1 of the first treatment if a chest X-ray or CT scan is not available within 6 months prior to that day.
5. History of sensitivity to heparin, heparin-induced thrombocytopenia, or sensitivity to any of the study medications, or components thereof, known allergy or hypersensitivity to milk protein or the excipients lactose monohydrate and magnesium stearate (MgSt), or a history of drug or other allergy that, in the opinion of the investigator or GSK Medical Monitor, contraindicates their participation.
6. The subject has taken prescription or non-prescription drugs, including CYP3A/PGP inhibitor, vitamins, herbal and dietary supplements (including St John's Wort) within 7 days (or 14 days if the drug is a potential enzyme inducer) or 5 half-lives (whichever is longer) prior to the first dose of study medication, unless in the opinion of the Investigator and Sponsor the medication will not interfere with the study procedures or compromise subject safety.
7. Positive result of urine cotinine test.
8. The subject has a history of cholecystectomy or biliary tract disease.

9. The subject has a significant clinical history or current conditions of glaucoma.
10. The subject has a significant clinical history or current conditions of prostatic hypertrophy.
11. The subject has a positive pre-study drug screen. A minimum list of drugs that were screened for included amphetamines, barbiturates, cocaine, opiates and benzodiazepines. The detection of drugs with a legitimate medical use was not necessarily an exclusion to study participation. The detection of alcohol was not an exclusion at screening but had to be negative pre-dose and during the study.
12. History of regular alcohol consumption within 3 months of the study defined as:

- Abuse of an average weekly intake of greater than 21 units or an average daily intake of greater than three units (males), or defined as an average weekly intake of greater than 14 units or an average daily intake of greater than two units (females). One unit was equivalent to a half-pint (220 mL) of beer or one (25 mL) measure of spirits or one glass (125 mL) of wine.

13. Female subjects, who are pregnant, planned pregnancy or lactation.
14. The subject has participated in a clinical trial and has received an investigational product within the following time period prior to the first dosing day in the current study: 30 days, 5 half-lives or twice the duration of the biological effect of the investigational product (whichever is longer).
15. Blood donation or sampled as a study subject within three months preceding the first dose of study drug and blood donation during the entire study in excess of 500mL.
16. A positive pre-study Hepatitis B surface antigen or positive Hepatitis C antibody result within 3 months of screening.
17. The subject has tested positive for HIV antibodies.
18. Unwillingness or inability to follow the procedures outlined in the protocol.
19. Subject is mentally or legally incapacitated.

5.2.3. Other Eligibility Criteria Considerations

To assess any potential impact on subject eligibility with regard to safety, the investigator must refer to the following document(s) for detailed information regarding warnings, precautions, contraindications, adverse events, and other significant data pertaining to the investigational product(s) being used in this study:

- GSK573719+GW642444 IB [GlaxoSmithKline Document Number RM2009/00437/02].
- GSK573719 IB [GlaxoSmithKline Document Number RM2006/00835/08].
- GW642444 IB [GlaxoSmithKline Document Number SM2003/00028/09].

6. DATA ANALYSIS AND STATISTICAL CONSIDERATIONS

6.1. Hypotheses and Treatment Comparisons

The primary objective of this study is to evaluate the pharmacokinetics of UMEC (62.5µg and 125µg) and VI (25µg) administered as single and repeat inhaled doses and in combinations of UMEC/VI (62.5g/25µg) and UMEC/VI (125µg/25µg) in healthy Chinese subjects. No formal hypothesis tests are planned, and an estimation approach will be adopted.

The primary comparisons of interest between treatment regimens (see Section 4.1) include:

(1) Effect of co-administration of 25µg VI on the UMEC PK exposure (e.g. AUC and Cmax) following single and repeat doses of UMEC:

   a. Treatment A vs C on Day 1 and Day 10

   b. Treatment B vs D on Day 1 and Day 10

(2) Effect of co-administration of at 62.5µg or 125µg UMEC on the VI PK exposure following single and repeat doses of GSK642444:

   a. Treatment A vs E on Day 1 and Day 10

   b. Treatment B vs E on Day 1 and Day 10

Point estimates and corresponding 90% confidence intervals will be presented for each treatment comparison.

6.2. Sample Size Considerations

6.2.1. Sample Size Assumptions

There is no formal hypothesis testing in this estimation approach driven study. This study is to evaluate the pharmacokinetics of UMEC and VI as monotherapies and concurrently in healthy Chinese subjects for the first time. Sample size is driven by feasibility as well as the requirement from Chinese regulatory agency (SFDA). A total of 20 healthy subjects aged 18-45 years will be randomised into the study. In case the number of evaluable subjects in any treatment group is less than 10 due to early withdrawal, the decision to replace will be made at the discretion of the Sponsor and Investigator so as to ensure at least 10 evaluable subjects per treatment group.

6.2.2. Sample Size Sensitivity

The above sample size is chosen to meet SFDA's requirement for PK studies. No sample size sensitivity analysis will be performed.

6.2.3. Sample Size Re-estimation

No sample size re-estimation will be performed.

6.3. Data Analysis Considerations

6.3.1. Interim Analysis

No formal interim analysis is planned.

6.3.2. Final Analyses

6.3.2.1. Analysis Population

Safety population

All subjects who receive at least one dose of study medication will be included in the safety population.

PK population

The PK Population is defined as all subjects in the Safety Population for whom a PK sample was obtained and analyzed.

6.3.2.2. Pharmacokinetic Analyses

6.3.2.2.1. Raw Plasma Concentrations

Linear and semi-logarithmic individual plasma concentration-time profiles and mean and median profiles of UMEC and VI by treatment group and day (Day 1 and Day 10) will be plotted. Plasma concentrations of UMEC and VI will be listed and summarized by treatment, day and nominal time, according to the PK Population.

Plasma concentration time data for UMEC and VI will be analyzed by non-compartmental methods with WinNonlin [version 4.1 or higher]. Calculations will be based on the actual sampling times recorded during the study.

6.3.2.2.2. Derived Plasma Pharmacokinetic Parameters

From the concentration-time data, the following pharmacokinetic parameters will be determined for each single dose of UMEC and VI: C_{max}, t_{max}, t_{last}, and AUC_{0-t}. Other parameters such as $t_{1/2}$, $AUC_{0-\infty}$, CL/F, Vd/F may be derived if data permits. If $AUC_{0-\infty}$ is noncalculable, $AUC_{0-t'}$ will be derived with t' being the latest time at which concentration is >NQ within each analyte across all subjects and treatments (for example AUC_{0-4} or AUC_{0-8} etc).

The following pharmacokinetic parameters will be determined from the plasma concentration-time data following repeated doses of UMEC and VI: C_{max}, t_{max}, t_{last}, AUC_{0-t}, and as data permit: terminal phase half life ($t_{½}$), C_{τ}, $AUC_{0-\tau}$, Ro and RC_{max}, DF. If $AUC_{0-\tau}$ is noncalculable, $AUC_{0-t'}$ will be derived with t' being the latest time at which concentration is >NQ within each analyte across all subjects and treatments (for example AUC_{0-4} or AUC_{0-8} etc).

If C_{max} is not calculable, it will be imputed with ½ the lower limit of quantification prior to calculating summary statistics. If AUC parameters are not calculable, they will be imputed with ½ the lowest calculable AUC within each analyte across all subjects and treatments. None of the other parameters will be imputed.

All derived pharmacokinetic parameters will be listed and summarized by treatment and day according to the PK Population.

6.3.2.2.3. *Steady State*

A plot of median trough plasma UMEC and VI concentrations on Day 6 to Day 10, as well as 24h post dose on Day 10 (Day 11) by treatment group (Linear and semi-log) and a plot of individual trough plasma UMEC and VI concentrations will be produced to visually inspect when steady state is achieved.

To formally evaluate whether steady state is achieved, statistical analysis of pre-dose concentration levels after log-transformation of the data on Day 6 to Day 10 as well as 24 hours post dose on Day 10 (Day 11) will be conducted for UMEC and VI separately. A mixed effect ANOVA model will be fit with treatment, day (as a continuous covariate) and treatment*day as a fixed effect and subject as a random effect for each analyte separately. The slope coefficient and the associated 90% confidence intervals will be derived to evaluate whether steady state is achieved.

6.3.2.2.4. *Assessment of accumulation*

Ro (the observed accumulation ratio calculated as $AUC_{0-\tau}$ on day 10 divided by $AUC_{0-\tau}$ on day 1) and RC_{max} (the accumulation ratio calculated as C_{max} on day 10 divided by C_{max} on day 1) will be derived for each subject.

Scatter plots of AUC $_{0-\tau}$ and C_{max} against day (Day 1 and 10) will be produced. The data points for each subject will be joined with straight lines. Each analyte will be put on one page so as to allow visual comparison of monotherapy with combination.

If $AUC_{0-\tau}$ is not calculable due to NQ data in terminal phase, $AUC_{0-t'}$ will be used with t' being the latest time at which concentration is >NQ for all subjects and days (Day 1 and Day 10) for a given treatment groups within analyte (for example AUC_{0-4} or AUC_{0-6} etc).

A formal assessment of accumulation may be performed for UMEC and VI separately through the statistical analysis of individual $AUC_{0-\tau}$ and C_{max} after log-transformation of the data on Day 1 and Day 10. A mixed effect model in line with GSK SOP-CPK-0007 will be fitted to estimate treatment effect. More details will be provided in the RAP.

6.3.2.2.5. Time-Invariance

Rs (steady state ratio calculated as $AUC_{0-\tau}$ on Day 10 divided by $AUC_{0-\infty}$ on Day1) will be derived for each subject and will be evaluated in a similar manner as assessment of accumulation if data permits.

6.3.2.2.6. Treatment Comparison of Systemic Exposure

Systemic exposure of VI and UMEC will be evaluated when administered in combination compared to administration alone using loge- transformed $AUC_{0-\infty}$ and C_{max} in a mixed effects model in line with SOP-BMD-4002 by analyte and by parameter. If $AUC_{0-\infty}$ is noncalculable, $AUC_{0-t'}$ as defined in Section 6.3.2.2.2 may be used instead. More details will be provided in the RAP.

6.3.2.3. Pharmacokinetic/Pharmacodynamic Analyses

The relationship between individual maximum and weighted mean heart rate and QTcF, and VI and UMEC systemic exposure (C_{max}), will be explored graphically separately by single dose and repeated dose. If a relationship is observed this will be evaluated further with PK/PD model.

6.3.2.4. Safety Analyses

Full details of data analyses of safety in the safety population will be presented in Reporting and Analysis Plan (RAP).

The safety data of the safety population will be summarised descriptively and no formal statistical tests will be performed. Summaries of safety data will include adverse events, serious adverse events, vital signs, ECG (weighted mean (0-4h), Maximum value (0-4 hour) and any laboratory data. All safety data will be reported according to the specific treatment the subject received.

7. STUDY ASSESSMENTS AND PROCEDURES

This section lists the parameters of each planned study assessment. The exact timing of each assessment is listed in the Time and Events Tables (Section 4.6). Detailed procedures for obtaining each assessment are provided in the Study Procedures Manual (SPM).

Whenever vital signs, 12-lead ECGs and blood draws are scheduled for the same nominal time, the assessments should occur in the following order: 12-lead ECG, vital signs, blood draws.

Prior to being enrolled in the study and any study procedures being performed, subjects must sign the informed consent.

Each subject will undergo screening procedures within 21 days of the administration of their first dose of study medication and will have a follow up visit between 7 to 10 days inclusive post their last dose. See Section 4.6.for assessments performed at the screening and follow-up visits.

The timing and number of planned study assessments including safety or pharmacokinetic assessments may be altered during the course of the study based on newly available data (e.g. to obtain data closer to the time of peak plasma concentrations) to ensure appropriate monitoring. The change in timing or addition of time points for any planned study assessments must be approved and documented by GSK, but this will not constitute a protocol amendment. The IRB/IEC will be informed of any safety issues that require alteration of the safety monitoring scheme.

No more than 350 mL of blood will be collected over the duration of the study, including any extra assessments that may be required.

7.1. Demographic/Medical History Assessments

The following demographic parameters will be captured: date of birth, gender, race and ethnicity.

Medical/medication/alcohol history will be assessed as related to the eligibility criteria listed in Section 5.2.

7.2. Safety

Planned time points for all safety assessments are listed in the Time and Events Table (Section 4.6). Additional time points for safety tests (such as vital signs, physical exams and laboratory safety tests) may be added during the course of the study based on newly available data to ensure appropriate safety monitoring.

Physical Exams

- A complete physical examination will include assessments of the head, eyes, ears, nose, throat, skin, thyroid, neurological, lungs, cardiovascular, abdomen (liver and spleen), lymph nodes and extremities. Height and weight will also be measured and recorded.
- A brief physical examination will include assessments of the skin, lungs, cardiovascular system, and abdomen (liver and spleen).

Vital Signs

- Vital sign measurements will include systolic and diastolic blood pressure and pulse rate.
- Resting heart rate and blood pressure measurements will be made at screening, at various occasions throughout the treatment periods, and, at the follow-up visit as outlined in Section 4.6 Time and Events Table.
- Three measurements will be taken at screening, 1 – 5mins apart. The mean value recorded will be classed as baseline.
- Subject will be required to rest in the sit position for at least 5mins before each reading.

Electrocardiogram (ECG)

- 12-lead ECGs will be obtained at each timepoint during the study using an ECG machine that calculates the heart rate and measures PR, QRS, QT, and QTcF intervals. Refer to Section 4.5.2 for QTcF withdrawal criteria and additional QTcF readings that may be necessary.
- Twelve-lead ECGs will be performed at screening, at various occasions throughout the treatment periods and at the follow-up visit as outlined in Section 4.6. Time and Events Table.
- Three measurements will be taken at screening and pre-dose, 1-5mins apart. The mean value recorded pre-dose will be classed as baseline.
- ECG measurements will be made (1-5mins apart for repeat measurements) with the subject in a supine having rested in this position for at least 5 mins before each reading.
- ECG-related stopping criteria for subjects are described in Section 4.5.2.

Clinical Laboratory Assessments

Hematology, clinical chemistry, urinalysis and additional parameters to be tested are listed below:

Hematology

Platelet Count	*RBC Indices*:	*Automated WBC Differential:*
RBC Count	MCV	Neutrophils
WBC Count (absolute)	MCH	Lymphocytes
Hemoglobin	MCHC	Monocytes
Hematocrit		Eosinophils
		Basophils

Clinical Chemistry

BUN	Potassium	AST (GOT)	Total and direct bilirubin
Creatinine	Calcium	ALT (GPT)	Uric Acid
Glucose, fasting		GGT	Albumin
Sodium		Alkaline phosphatase	Total Protein

Routine Urinalysis

Specific gravity
pH, glucose, protein, blood and ketones by dipstick
Microscopic examination (if blood or protein is abnormal)

Other screening tests

HIV
Hepatitis B (HBsAg)
Hepatitis C (Hep C antibody)
FSH and estradiol (as needed in women of non-child bearing potential only)
Alcohol and urine cotinine screen

Further details regarding these tests can be obtained from the SPM.

7.3. Pregnancy

7.3.1. Time period for collecting pregnancy information

All pregnancies in female subjects and/or female partners of male subjects will be collected after the start of dosing and until follow-up visit.

7.3.2. Action to be taken if pregnancy occurs

The investigator will collect pregnancy information on any female subject, who becomes pregnant while participating in this study. The investigator will record pregnancy information on the appropriate form and submit it to GSK within 2 weeks of learning of a

subject's pregnancy. The subject will also be followed to determine the outcome of the pregnancy. Information on the status of the mother and child will be forwarded to GSK. Generally, follow-up will be no longer than 6 to 8 weeks following the estimated delivery date. Any premature termination of the pregnancy will be reported.

While pregnancy itself is not considered to be an AE or SAE, any pregnancy complication or elective termination of a pregnancy for medical reasons will be recorded as an AE or SAE.

A spontaneous abortion is always considered to be an SAE and will be reported as such. Furthermore, any SAE occurring as a result of a post-study pregnancy and is considered reasonably related to the study treatment by the investigator, will be reported to GSK as described in Section 12 . While the investigator is not obligated to actively seek this information in former study participants, he or she may learn of an SAE through spontaneous reporting.

Any female subject who becomes pregnant while participating will be withdrawn from the study

7.4. Pharmacokinetics

7.4.1. Blood Sample Collection

Blood samples for pharmacokinetic analysis of UMEC and VI will be collected at the time points indicated in Section 4.6, Time and Events Table. The actual date and time of each blood sample collection will be recorded. The timing of PK samples may be altered and/or PK samples may be obtained at additional time points to ensure thorough PK monitoring.

Details of PK blood sample collection (including volume to be collected), processing, storage and shipping procedures are provided in the Study Procedures Manual (SPM).

7.4.2. Sample Analysis

Concentrations of UMEC and VI will be determined in plasma using the currently approved analytical methodology.

8. LIFESTYLE AND/OR DIETARY RESTRICTIONS

8.1. Contraception Requirements

8.1.1. Female Subjects

Female subjects of childbearing potential must not become pregnant and so must be sexually inactive by abstinence or use contraceptive methods with a failure rate of < 1%.

Abstinence

Sexual inactivity by abstinence must be consistent with the preferred and usual lifestyle of the subject. Periodic abstinence (e.g. calendar, ovulation, symptothermal, post-ovulation methods) and withdrawal are not acceptable methods of contraception.

Contraceptive Methods with a Failure Rate of < 1%

- Oral contraceptive, either combined or progestogen alone
- Injectable progestogen
- Implants of levonorgestrel
- Estrogenic vaginal ring
- Percutaneous contraceptive patches
- Intrauterine device (IUD) or intrauterine system (IUS) that meets the <1% failure rate as stated in the product label
- Male partner sterilization (vasectomy with documentation of azoospermia) prior to the female subject's entry into the study, and this male is the sole partner for that subject. For this definition, "documented" refers to the outcome of the investigator's/designee's medical examination of the subject or review of the subject's medical history for study eligibility, as obtained via a verbal interview with the subject or from the subject's medical records.
- Double barrier method: condom and occlusive cap (diaphragm or cervical/vault caps) plus spermicidal agent (foam/gel/film/cream/suppository)

These allowed methods of contraception are only effective when used consistently, correctly and in accordance with the product label. The investigator is responsible for ensuring subjects understand how to properly use these methods of contraception.

8.1.2. Male Subjects

Male subjects with female partners of child-bearing potential must use one of the following contraceptive methods after the first dose of study treatment and until the follow-up contact:

- Condom plus partner use of a highly effective contraceptive such as occlusive cap (diaphragm or cervical/vault cap) plus spermicidal agent (foam/gel/film/cream/suppository), oral contraceptive, injectable progesterone, implant of etonogestrel or levonorgestrel, estrogenic vaginal ring, percutaneous contraceptive patches, or intrauterine device. **OR**
- Abstinence, defined as sexual inactivity consistent with the preferred and usual lifestyle of the subject. Periodic abstinence (e.g. calendar, ovulation, symptothermal, post-ovulation methods) and withdrawal are not acceptable methods of contraception

8.2. Meals and Dietary Restrictions

- Subjects will be required to follow the fasting restrictions described below:

Treatment period	Morning
Day 1, 10	Fast: 4hr predose – 4hr post dose
Day 6,7,8,9	Fast: 2hr predose – 1hr post dose

- Water will be allowed freely, except for 1hour either side of dosing on all dosing occasions.
- Subjects must refrain from grapefruit or grapefruit juice containing products from 7 days pre-dose (Day 1) of the first treatment period until collection of the final PK blood sample. Subsequent meals and snacks will be standardized for all subjects during their in-site period.

8.3. Caffeine, Alcohol, and Tobacco

- During each dosing session, subjects will abstain from ingesting caffeine- or xanthine-containing products (e.g. coffee, tea, cola drinks, and chocolate) for 24 hours prior to the start of dosing until collection of the final pharmacokinetic and or pharmacodynamic sample during each session.
- During each dosing session, subjects will abstain from alcohol for 24 hours prior to the start of dosing until collection of the final pharmacokinetic and or pharmacodynamic sample during each session.
- Use of tobacco products is not allowed from screening until after the final follow-up visit.

8.4. Activity

Subjects will abstain from strenuous exercise for 48 hours prior to each blood collection for clinical laboratory tests. Subjects may participate in light recreational activities during studies (e.g., watch television, read). Strong activities and long time bed rest should be avoided.

9. CONCOMITANT MEDICATIONS AND NON-DRUG THERAPIES

9.1. Permitted Medications

Subjects may take simple analgesics, such as paracetamol/acetaminophen, aspirin, ibuprofen, and et al, following the prescription of professional physician.

9.2. Prohibited Medications

Other than simple analgesics, such as paracetamol/acetaminophen, aspirin, ibuprofen, and et al , subjects must abstain from taking prescription or non-prescription drugs (including vitamins and dietary or herbal supplements), within 7 days (or 14 days if the drug is a potential enzyme inducer) or 5 half-lives (whichever is longer) prior to the first dose of study medication until completion of the follow-up visit, unless in the opinion of the Investigator and sponsor the medication will not interfere with the study.

The Investigator (or designated study physician) must be informed as soon as possible about any medication taken from the time of screening until the end of the clinical phase of the study (follow-up visit).

All concomitant medications taken during the study will be recorded in the CRF. The minimum requirement is that drug name and the dates of administration are to be recorded.

9.3. Non-Drug Therapies

The Investigator (or designated study physician) must be informed as soon as possible about any non-drug therapies from the time of screening until the end of the clinical phase of the study (follow-up visit).

10. COMPLETION OR EARLY WITHDRAWAL OF SUBJECTS

10.1. Subject Completion

A completed subject is one who has completed all phases of the study including the follow-up visit.

The end of the study is defined as the last subject's last visit.

10.2. Subject Withdrawal Criteria

Refer to Section 4.5 for dose adjustment/stopping criteria based on safety/PK/PD criteria.

A subject may withdraw from study treatment at any time at his/her own request, or may be withdrawn at any time at the discretion of the investigator for safety, behavioral or administrative reasons.

10.3. Subject Withdrawal Procedures

10.3.1. Subject Withdrawal from Study

For subjects withdrawn from the investigational product, follow-up procedures should be performed. Relevant pages of the CRF should be completed by the investigator

10.4. Treatment after the End of the Study

Subjects will not receive any additional treatment after completion of the study because only healthy volunteers are eligible for study participation.

10.5. Screen and Baseline Failures

Data for screen and baseline failures will be collected in source documentation at the site but will not be transmitted to GSK. The reason(s) for excluding these subjects will be documented. The source documentation (including ICF) for all such failures will be made available to the monitor for monitoring.

11. MADE AVAILABLE TO THE MONITOR FOR MONITORING STUDY TREATMENT

Study treatment dosage and administration details are listed in Section 4.4.

11.1. Blinding

This will be an open label study.

11.2. Packaging and Labeling

The contents of the label will be in accordance with all applicable regulatory requirements.

11.3. Preparation/Handling/Storage/Accountability

No special preparation of study treatment is required.

Study treatment must be dispensed or administered according to procedures described herein. Only subjects enrolled in the study may receive study treatment. Only authorized site staff may supply or administer study treatment. All study treatment must be stored in a secure area with access limited to the investigator and authorized site staff. Study treatment is to be stored at physical conditions consistent with investigational product-specific requirements. Maintenance of a temperature log (manual or automated) is required.

The investigator, institution, or the head of the medical institution (where applicable) is responsible for study treatment accountability, reconciliation, and record maintenance. The investigator or the head of the medical institution (where applicable), or designated site staff (e.g., storage manager, where applicable) must maintain study treatment accountability records throughout the course of the study. The responsible person(s) will document the amount of study treatment received from and returned to GSK and the amount supplied and/or administered to and/or returned by subjects. The required accountability unit for this study will be inhalers. Discrepancies are to be reconciled or

resolved. Procedures for final disposition of unused study treatment are listed in the SPM.

Investigational product is not expected to pose significant occupational safety risk to site staff under normal conditions of use and administration. A Material Safety Data Sheet (MSDS)/equivalent document describing occupational hazards and recommended handling precautions either will be provided to the investigator, where this is required by local laws, or is available upon request from GSK.

However, precautions are to be taken to avoid direct skin contact, eye contact, and generating aerosols or mists. In the case of unintentional occupational exposure notify the monitor, medical monitor and/or study manager.

Precaution will be taken to avoid direct contact with the investigational product. A Material Safety Data Sheet (MSDS) describing occupational hazards and recommended handling precautions will be provided to the investigator.

11.4. Assessment of Compliance

When subjects are dosed at the study site, they will receive study treatment directly from the investigator or designee, under medical supervision. The date and time of each dose administered in the clinic will be recorded in the source documents. The dose of study treatment and study participant identification will be confirmed at the time of dosing by a member of the study site staff other than the person administering the study treatment. Study site personnel will examine the counter on the subject's novel dry powder inhaler to ensure that the investigational product was dispensed.

11.5. Treatment of Study Treatment Overdose

An overdose for this study will be considered as any dose of study drug more than the planned dose on each dosing occasion. In the event of an overdose, there are no recommended medications or non-drug therapies for treatment. Management should be supportive and the investigator should use his/her clinical judgment in treating any overdose situation. Subjects experiencing such adverse events will be followed up clinically until the event has resolved.

GSK does not recommend specific treatment for an overdose. The investigator will use clinical judgment to treat any overdose.

12. ADVERSE EVENTS (AE) AND SERIOUS ADVERSE EVENTS (SAE)

The investigator or site staff is responsible for detecting, documenting and reporting events that meet the definition of an AE or SAE.

All AEs will be collected from the start of study treatment until the follow-up contact. Medical occurrences that begin prior to the start of study treatment but after obtaining

informed consent may be recorded on the Medical History/Current Medical Conditions CRF.

Serious AEs will be recorded from the time the consent form is signed until the follow-up contact. All SAEs will be recorded and reported to GSK within 24 hours, as indicated in Section 12.7.

Investigators are not obligated to actively seek AEs or SAEs in former study participants. However, if the investigator learns of any SAE, including a death, at any time after a subject has been discharged from the study, and he/she considers the event reasonably related to the study treatment or study participation, the investigator would promptly notify GSK.

12.1. Definition of Adverse Events

An AE is any untoward medical occurrence in a patient or clinical investigation subject, temporally associated with the use of a medicinal product, whether or not considered related to the medicinal product.

Note: An AE can therefore be any unfavorable and unintended sign (including an abnormal laboratory finding), symptom, or disease (new or exacerbated) temporally associated with the use of a medicinal product.

Events meeting the definition of an AE **include**:

- Any abnormal laboratory test results (hematology, clinical chemistry, or urinalysis) or other safety assessments (e.g., ECGs, radiological scans, vital signs measurements), including those that worsen from baseline, and felt to be clinically significant in the medical and scientific judgment of the investigator.
- Exacerbation of a chronic or intermittent pre-existing condition including either an increase in frequency and/or intensity of the condition.
- New conditions detected or diagnosed after study treatment administration even though it may have been present prior to the start of the study.
- Signs, symptoms, or the clinical sequelae of a suspected interaction.
- Signs, symptoms, or the clinical sequelae of a suspected overdose of either study treatment or a concomitant medication (overdose per se will not be reported as an AE/SAE).

Events that **do not** meet the definition of an AE include:

- Any clinically significant abnormal laboratory findings or other abnormal safety assessments that are associated with the underlying disease, unless judged by the investigator to be more severe than expected for the subject's condition.
- The disease/disorder being studied, or expected progression, signs, or symptoms of the disease/disorder being studied, unless more severe than expected for the subject's condition.
- Medical or surgical procedure (e.g., endoscopy, appendectomy); the condition that leads to the procedure is an AE.
- Situations where an untoward medical occurrence did not occur (social and/or convenience admission to a hospital).
- Anticipated day-to-day fluctuations of pre-existing disease(s) or condition(s) present or detected at the start of the study that do not worsen.

12.2. Definition of Serious Adverse Events

If an event is not an AE per Section 12.1, then it cannot be an SAE even if serious conditions are met (e.g., hospitalization for signs/symptoms of the disease under study, death due to progression of disease, etc).

An SAE is any untoward medical occurrence that, at any dose:

a. Results in death

b. Is life-threatening

NOTE: The term 'life-threatening' in the definition of 'serious' refers to an event in which the subject was at risk of death at the time of the event. It does not refer to an event, which hypothetically might have caused death, if it were more severe.

c. Requires hospitalization or prolongation of existing hospitalization

NOTE: In general, hospitalization signifies that the subject has been detained (usually involving at least an overnight stay) at the hospital or emergency ward for observation and/or treatment that would not have been appropriate in the physician's office or outpatient setting. Complications that occur during hospitalization are AEs. If a complication prolongs hospitalization or fulfills any other serious criteria, the event is serious. When in doubt as to whether "hospitalization" occurred or was necessary, the AE should be considered serious.

Hospitalization for elective treatment of a pre-existing condition that did not worsen from baseline is not considered an AE.

d. Results in disability/incapacity, or

NOTE: The term disability means a substantial disruption of a person's ability to conduct normal life functions. This definition is not intended to include experiences of relatively minor medical significance such as uncomplicated headache, nausea, vomiting, diarrhea, influenza, and accidental trauma (e.g. sprained ankle) which may interfere or prevent everyday life functions but do not constitute a substantial disruption.

e. Is a congenital anomaly/birth defect

f. Medical or scientific judgment should be exercised in deciding whether reporting is appropriate in other situations, such as important medical events that may not be immediately life-threatening or result in death or hospitalization but may jeopardize the subject or may require medical or surgical intervention to prevent one of the other outcomes listed in the above definition. These should also be considered serious. Examples of such events are invasive or malignant cancers, intensive treatment in an emergency room or at home for allergic bronchospasm, blood dyscrasias or convulsions that do not result in hospitalization, or development of drug dependency or drug abuse.

g. Is associated with liver injury **and** impaired liver function defined as:

- ALT ≥ 3xULN, and
- Total bilirubin ≥ 2xULN or INR > 1.5.

NOTES:

Bilirubin fractionation should be performed if testing is available. If fractionation is unavailable, urinary bilirubin is to be measured via dipstick (a measurement of direct bilirubin, which would suggest liver injury).

INR measurement is not required; if measured, the threshold value stated will not apply to patients receiving anticoagulants.

If INR measurement is obtained, the value is to be recorded on the SAE form.

12.3. Method of Detecting AEs and SAEs

Care will be taken not to introduce bias when detecting AEs and/or SAEs. Open-ended and non-leading verbal questioning of the subject is the preferred method to inquire about AE occurrence. Appropriate questions include:

- "How are you feeling
- "Have you had any (other) medical problems since your last visit/contact?"
- "Have you taken any new medicines, other than those provided in this study, since your last visit/contact?"

12.4. Recording of AEs and SAEs

When an AE/SAE occurs, it is the responsibility of the investigator to review all documentation (e.g., hospital progress notes, laboratory, and diagnostics reports) relative to the event. The investigator will then record all relevant information regarding an AE/SAE in the appropriate data collection tool.

It is not acceptable for the investigator to send photocopies of the subject's medical records to GSK in lieu of completion of the GSK, AE/SAE data collection tool. However, there may be instances when copies of medical records for certain cases are requested by GSK. In this instance, all subject identifiers, with the exception of the subject number, will be blinded on the copies of the medical records prior to submission of to GSK.

The investigator will attempt to establish a diagnosis of the event based on signs, symptoms, and/or other clinical information. In such cases, the diagnosis will be documented as the AE/SAE and not the individual signs/symptoms.

12.5. Evaluating AEs and SAEs

12.5.1. Assessment of Intensity

The investigator will make an assessment of intensity for each AE and SAE reported during the study and will assign it to one of the following categories:

Mild: An event that is easily tolerated by the subject, causing minimal discomfort and not interfering with everyday activities.

Moderate: An event that is sufficiently discomforting to interfere with normal everyday activities.

Severe: An event that prevents normal everyday activities.

An AE that is assessed as severe will not be confused with an SAE. Severity is a category utilized for rating the intensity of an event; and both AEs and SAEs can be assessed as severe. An event is defined as 'serious' when it meets at least one of the pre-defined outcomes as described in the definition of an SAE.

12.5.2. Assessment of Causality

The investigator is obligated to assess the relationship between study treatment and the occurrence of each AE/SAE. A "reasonable possibility" is meant to convey that there are facts/evidence or arguments to suggest a causal relationship, rather than a relationship cannot be ruled out. The investigator will use clinical judgment to determine the relationship. Alternative causes, such as natural history of the underlying diseases, concomitant therapy, other risk factors, and the temporal relationship of the event to the study treatment will be considered and investigated. The investigator will also consult the Investigator Brochure (IB) and/or Product Information, for marketed products, in the determination of his/her assessment.

There may be situations when an SAE has occurred and the investigator has minimal information to include in the initial report to GSK. However, **it is very important that the investigator always make an assessment of causality for every event prior to the initial transmission of the SAE data to GSK.** The investigator may change his/her opinion of causality in light of follow-up information, amending the SAE data collection tool accordingly. The causality assessment is one of the criteria used when determining regulatory reporting requirements.

12.6. Follow-up of AEs and SAEs

After the initial AE/SAE report, the investigator is required to proactively follow each subject at subsequent visits/contacts. All AEs and SAEs will be followed until resolution, until the condition stabilizes, until the event is otherwise explained, or until the subject is lost to follow-up.

The investigator is obligated to perform or arrange for the conduct of supplemental measurements and/or evaluations as may be indicated or as requested by GSK to elucidate as fully as possible the nature and/or causality of the AE or SAE. The investigator is obligated to assist. This may include additional laboratory tests or investigations, histopathological examinations or consultation with other health care professionals. If a subject dies during participation in the study or during a recognized follow-up period, the investigator will provide GSK with a copy of any post-mortem findings, including histopathology.

New or updated information will be recorded in the originally completed data collection tool. The investigator will submit any updated SAE data to GSK within the designated reporting time frames.

12.7. Prompt Reporting of SAEs to GSK

Once the investigator determines that an event meets the protocol definition of an SAE, the SAE will be reported to GSK **within 24 hours**. Any follow-up information on a previously reported SAE will also be reported to GSK within 24 hours.

If the investigator does not have all information regarding an SAE, he/she will not wait to receive additional information before notifying GSK of the event and completing the appropriate data collection tool. The investigator will always provide an assessment of causality at the time of the initial report as described in Section 12.5.2, Assessment of Causality.

The primary mechanism for reporting SAEs to GSK will be using the paper SAE data collection tool and fax it to the GSK Medical Monitor Dr [redacted].

After the study is completed at a given site, if the site receives a report of a new SAE from a study participant or receives updated data on a previously reported SAE after the electronic data collection tool has been taken off-line, the site can report this information on a paper SAE form or to their GSK protocol contact by telephone.

GSK contacts for SAE receipt can be found at the beginning of this protocol on the Sponsor/Medical Monitor Contact Information page.

12.8. Regulatory Reporting Requirements for SAEs

Prompt notification of SAEs by the Investigator to GlaxoSmithKline is essential so that legal obligations and ethical responsibilities towards the safety of patients are met.

The investigator should report SAE/Serious Incident to SFDA/GSK within 24hours timeframe from the initial and follow-up notification of the SAE/Serious Incident, and report to site Ethics Committee (EC) based on local EC requirement.

GlaxoSmithKline has a legal responsibility to notify both the local regulatory authority and other regulatory agencies about the safety of a product under clinical investigation. GlaxoSmithKline will comply with country specific regulatory requirements relating to safety reporting to the regulatory authority, Independent Ethics Committee (IEC) and Investigators.

The NSC is responsible for individual expedited reports submission to SFDA within 15 days from GSK first receipt (7 days for death case).

Investigator safety reports are prepared for suspected unexpected serious adverse reactions according to local regulatory requirements and GlaxoSmithKline policy and are forwarded to Investigators as necessary.

An Investigator who receives an Investigator safety report describing a SAE(s) or other specific safety information (e.g., summary or listing of SAEs) from GlaxoSmithKline will file it with the IB and will notify the IEC, if appropriate according to local requirements.

13. LIVER CHEMISTRY FOLLOW-UP PROCEDURES

Refer to the diagram in Appendix 1 for a visual presentation of the procedures listed below.

The procedures listed below are to be followed if a subject meets the liver chemistry stopping criteria defined in Section 4.5.1:

- Immediately and permanently withdraw the subject from study treatment
- Notify the GSK medical monitor within 24 hours of learning of the abnormality to confirm the subject's study treatment cessation and follow-up.
- Complete the "Safety Follow-Up Procedures" listed below.
- Complete the liver event case report forms. If the event also meets the criteria of an SAE (see Section 12.2), the SAE data collection tool will be completed separately with the relevant details.
- Upon completion of the safety follow-up permanently withdraw the subject from the study and do not rechallenge with study treatment.

Safety Follow-Up Procedures for subjects with ALT ≥ 3xULN:

- Monitor subjects weekly until liver chemistries (ALT, AST, alkaline phosphatase, bilirubin) resolve, stabilize or return to within baseline values.

Safety Follow-Up Procedures for subjects with ALT ≥3xULN and bilirubin ≥2xULN (or ALT ≥ 3xULN and INR[1] > 1.5):

- This event is considered an SAE (see Section 12.2). Serum bilirubin fractionation should be performed if testing is available. If fractionation is unavailable, urinary bilirubin is to be measured via dipstick (a measurement of direct bilirubin, which would suggest liver injury).
- Make every reasonable attempt to have subjects return to the clinic within 24 hours for repeat liver chemistries, additional testing, and close monitoring (with specialist or hepatology consultation recommended).
- Monitor subjects twice weekly until liver chemistries (ALT, AST, alkaline phosphatase, bilirubin) resolve, stabilize or return to within baseline values.

In addition, for all subjects with ALT ≥ 3xULN, every attempt must be made to also obtain the following:

- Viral hepatitis serology including:
  - Hepatitis A IgM antibody.
  - Hepatitis B surface antigen and Hepatitis B Core Antibody (IgM).
  - Hepatitis C RNA.
  - Cytomegalovirus IgM antibody.
  - Epstein-Barr viral capsid antigen IgM antibody (or if unavailable, obtain heterophile antibody or monospot testing).
  - Hepatitis E IgM antibody.
- Blood sample for pharmacokinetic (PK) analysis, obtained within 24 hours or 3 half lives of last dose of last dose. Record the date/time of the PK blood sample draw and the date/time of the last dose of study treatment prior to blood sample draw on the CRF. If the date or time of the last dose is unclear, provide the subject's best approximation. If the date/time of the last dose cannot be approximated OR a PK sample cannot be collected in the time period indicated above, do not obtain a PK sample. Instructions for sample handling and shipping are included in the SPM.
- Serum creatine phosphokinase (CPK) and lactate dehydrogenase (LDH).
- Fractionate bilirubin, if total bilirubin ≥ 2xULN.
- Assess eosinophilia

[1] INR testing not required per protocol and the threshold value does not apply to subjects receiving anticoagulants.

- Record the appearance or worsening of clinical symptoms of hepatitis (fatigue, nausea, vomiting, right upper quadrant pain or tenderness, fever, rash or eosinophilia) on the AE CRF.
- Record use of concomitant medications, acetaminophen, herbal remedies, other over the counter medications, or putative hepatotoxins on the Concomitant Medications CRF.
- Record alcohol use on the Liver Events CRF.

The following are required for subjects with ALT ≥ 3xULN **and** bilirubin ≥ 2xULN but are optional for other abnormal liver chemistries:

- Anti-nuclear antibody, anti-smooth muscle antibody, and Type 1 anti-liver kidney microsomal antibodies.
- Liver imaging (ultrasound, magnetic resonance, or computerized tomography) to evaluate liver disease.
- The Liver Imaging and/or Liver Biopsy CRFs are also to be completed if these tests are performed.

14. STUDY CONDUCT CONSIDERATIONS

14.1. Posting of Information on Clinicaltrials.gov

Study information from this protocol will be posted on clinicaltrials.gov before enrollment of subjects begins.

14.2. Regulatory and Ethical Considerations, Including the Informed Consent Process

GSK will obtain favorable opinion/approval to conduct the study from the appropriate regulatory agency in accordance with any applicable country-specific regulatory requirements prior to a site initiating the study in that country.

The study will be conducted in accordance with all applicable regulatory requirements.

The study will also be conducted in accordance with ICH Good Clinical Practice (GCP), all applicable subject privacy requirements, and, the guiding principles of the 2008 Declaration of Helsinki. This includes, but is not limited to, the following:

- IRB/IEC review and favorable opinion/approval to conduct the study and of any subsequent relevant amended documents
- Written informed consent (and any amendments) to be obtained for each subject before participation in the study
- Investigator reporting requirements (e.g. reporting of AEs/SAEs/protocol deviations to IRB/IEC)

14.2.1. Urgent Safety Measures

If an event occurs that is related to the conduct of the study or the development of the study treatment, and this new event is likely to affect the safety of subjects, the sponsor and the investigator will take appropriate urgent safety measures to protect subjects against any immediate hazard.

The sponsor will work with the investigator to ensure the IEC/IRB is notified.

14.3. Quality Control (Study Monitoring)

In accordance with applicable regulations including GCP, and GSK procedures, GSK monitors will contact the site prior to the start of the study to review with the site staff the protocol, study requirements, and their responsibilities to satisfy regulatory, ethical, and GSK requirements. When reviewing data collection procedures, the discussion will also include identification, agreement and documentation of data items for which the CRF will serve as the source document.

GSK will monitor the study and site activity to verify that the:

- Data are authentic, accurate, and complete.
- Safety and rights of subjects are being protected.
- Study is conducted in accordance with the currently approved protocol and any other study agreements, GCP, and all applicable regulatory requirements.

The investigator and the head of the medical institution (where applicable) agrees to allow the monitor direct access to all relevant documents

14.4. Quality Assurance

To ensure compliance with GCP and all applicable regulatory requirements, GSK may conduct a quality assurance audit. Regulatory agencies may also conduct a regulatory inspection of this study. Such audits/inspections can occur at any time during or after completion of the study. If an audit or inspection occurs, the investigator and institution agree to allow the auditor/inspector direct access to all relevant documents and to allocate his/her time and the time of his/her staff to the auditor/inspector to discuss findings and any relevant issues.

14.5. Study and Site Closure

Upon completion or premature discontinuation of the study, the monitor will conduct site closure activities with the investigator or site staff, as appropriate, in accordance with applicable regulations including GCP, and GSK procedures.

In addition, GSK reserves the right to temporarily suspend or prematurely discontinue this study at any time for reasons including, but not limited to, safety or ethical issues or severe non-compliance. If GSK determines such action is needed, GSK will discuss this

with the investigator or the head of the medical institution (where applicable), including the reasons for taking such action. When feasible, GSK will provide advance notification to the investigator or the head of the medical institution, where applicable, of the impending action prior to it taking effect.

If the study is suspended or prematurely discontinued for safety reasons, GSK will promptly inform investigators or the head of the medical institution (where applicable) and the regulatory authorities of the suspension or premature discontinuation of the study and the reason(s) for the action. If required by applicable regulations, the investigator or the head of the medical institution (where applicable) must inform the IRB/IEC promptly and provide the reason for the suspension or premature discontinuation.

14.6. Records Retention

Following closure of the study, the investigator or the head of the medical institution (where applicable) must maintain all site study records, except for those required by local regulations to be maintained by someone else, in a safe and secure location. The records must be maintained to allow easy and timely retrieval, when needed (e.g., audit or inspection), and, whenever feasible, to allow any subsequent review of data in conjunction with assessment of the facility, supporting systems, and staff. Where permitted by local laws/regulations or institutional policy, some or all of these records can be maintained in a format other than hard copy (e.g., microfiche, scanned, electronic); however, caution needs to be exercised before such action is taken. The investigator must assure that all reproductions are legible and are a true and accurate copy of the original, and meet accessibility and retrieval standards, including re-generating a hard copy, if required. Furthermore, the investigator must ensure there is an acceptable back-up of these reproductions and that an acceptable quality control process exists for making these reproductions.

According to ICH GCP and China GCP, all subject identification codes, subject files and Clinical Trial data and related documents will be kept by Institution in a secure place for a period of five (5) years after completion of the Clinical Trial. After the 5-year period, all documents kept by Institution will be transferred to the third party designated by GSK and kept in accordance with GSK policies. GSK will bear the archiving fee.

The investigator must notify GSK of any changes in the archival arrangements, including, but not limited to, archival at an off-site facility or transfer of ownership of the records in the event the investigator leaves the site.

14.7. Provision of Study Results to Investigators, Posting to the Clinical Trials Register and Publication

Where required by applicable regulatory requirements, an investigator signatory will be identified for the approval of the clinical study report. The investigator will be provided reasonable access to statistical tables, figures, and relevant reports and will have the opportunity to review the complete study results at a GSK site or other mutually-agreeable location.

GSK will also provide the investigator with the full summary of the study results. The investigator is encouraged to share the summary results with the study subjects, as appropriate.

GSK will provide the investigator with the randomization codes for their site only after completion of the full statistical analysis.

14.8. Data Management

GSK Data Management will identify and implement the most effective data acquisition and management strategy for each clinical trial protocol and deliver datasets which support the protocol objectives. Subject data will be entered into GSK defined CRFs and combined with data provided from other sources (e.g. diary data, laboratory data) in a validated data system. Subject initials will not be transmitted to GSK for inclusion in the datasets. Clinical data management will be performed in accordance with applicable GSK standards and data cleaning procedures with the objective of removing errors and inconsistencies in the data which would otherwise impact on the analysis and reporting objectives, or the credibility of the Clinical Study Report. Adverse events and concomitant medications terms will be coded using validated dictionaries. Original CRFs will be retained by GSK, while the investigator will retain a copy.

15. REFERENCES

GlaxoSmithKline Document Number RM2009/00437/02, GSK573719+GW642444 Investigator's Brochure, Effective Date 21-Dec-2010

GlaxoSmithKline Document Number SM2003/00028/09, Vilanterol (GW642444) Investigator's Brochure, Effective Date 08-Sep-2011

GlaxoSmithKline Document Number RM2006/00835/08, Umeclidinium (GSK573719) Investigator's Brochure, Effective Date 16-Feb-2012

GOLD (Global Initiative for Chronic Obstructive Lung Disease). Global strategy for the diagnosis, management, and prevention of chronic obstructive pulmonary disease – Updated 2011. www.goldcopd.org.

16. APPENDICES

16.1. Appendix 1: Liver Safety Algorithms

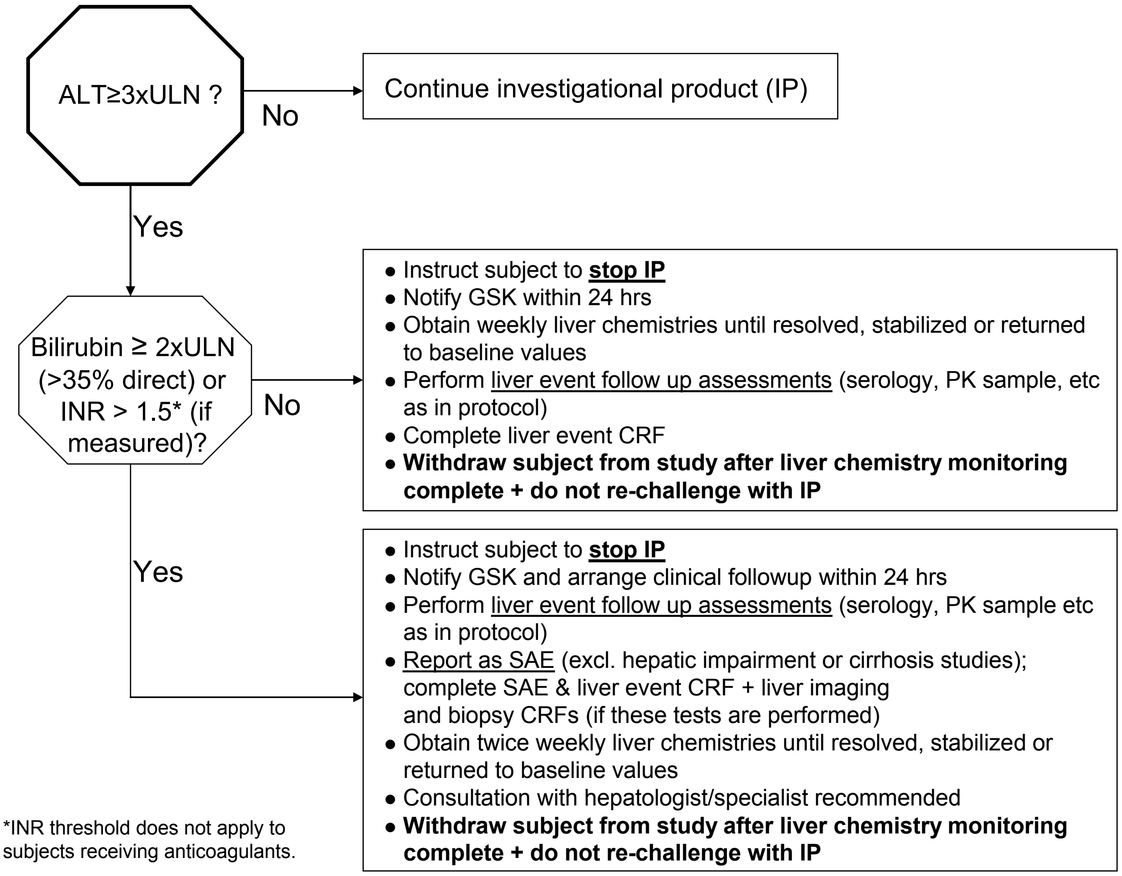

*INR threshold does not apply to subjects receiving anticoagulants.

16.2. Appendix 2: Amendment 1

The primary purpose of the amendment was to solve concerns from site staff on eligibility criteria, study assessment and procedures. In addition, several minor revisions to the protocol were made for consistency and accuracy.

The following is a detailed description of the changes that have been made to Protocol DB2115380, with additional text underlined.

Change #1

Change #1: To revise the time of PK sampling from ‘after dosing’ to ‘at designed timepoints’.

Reason for change #1: Keep consistent context. Blood PK sample will be collected at pre-dosing on D6-9 of treatment periods.

Description:

Original text:

Blood samples for PK analysis will be taken at regular intervals after dosing. Safety will be assessed by measurement of ECG QTcF, heart rate, blood pressure, and safety laboratory data and review of adverse events.

Amended text:

Blood samples for PK analysis will be taken at designed timepoints. Safety will be assessed by measurement of ECG QTcF, heart rate, blood pressure, and safety laboratory data and review of adverse events.

Change #2

Change #2: To update the sponsor staff list.

Reason for change #2: Medical monitor changed. Local PK expert is available.

Author(s):

Original text:

[redacted] (MDC, China); [redacted] (MDC, China); [redacted] (MDC, China); [redacted] (CPK M&S, US); [redacted] (MDC, US); [redacted] (CPMS, UK); [redacted] (Stat, UK).

Amended text:

[redacted] (MDC, China); [redacted] (MDC, China); [redacted] (MDC, China); [redacted] (MDC, China); [redacted] (CPK M&S, US); [redacted] (MDC, US); [redacted] (CPMS, UK); [redacted] (Stat, UK); [redacted] (MDC, China).

Sponsor/medical monitor Information Page

Original text:

**Role**	Name	Day Time Phone Number	After-hours Phone/Cell/ Pager Number	Fax Number	GSK Address
Primary Medical Monitor	[redacted]	[redacted]	[redacted]	[redacted]	No.1 Building, 917 Halei Road, Zhangjiang Hi-tech Park, Pudong Shanghai 201203, China

Amended text:

**Role**	Name	Day Time Phone Number	After-hours Phone/Cell/ Pager Number	Fax Number	GSK Address
Primary Medical Monitor	[redacted]	[redacted]	[redacted]	[redacted]	No.1 Building, 917 Halei Road, Zhangjiang Hi-tech Park, Pudong Shanghai 201203, China

Change #3

Change #3: To require the subjects to remain resident in site during the treatment periods.

Reason for change #3: To standardize the life style of subjects to obtain high-quality PK data.

4.1 Study Design/Schematic:

Original text:

Each subject will be admitted to the unit in the evening prior to Day 1 and remain resident until 8 hours post dosing; On day 2, 3, 4, 5, each subject will visit the unit for treatment administration; On day 6, 7, 8, 9, each subject will visit the unit for treatment administration and blood sampling; On Day 10 each subject will receive the last dosing and remain resident until all the 24 hour post dosing assessments have been completed, and get assessments at 48 hour after last dosing.

Amended text:

Each subject will be admitted to the unit in the evening prior to Day 1 and remain resident until all assessments completed on D12.

Change #4

Change #4: To update the date of laboratory safety test from D10 (12 hrs after morning dose) to D11.

Reason for change #4: To improve feasibility. By updating the timepoint, the subject can avoid keeping extra 8-hour fast for clinical chemistry test on D10.

4.6 Time and Events Table:

Original text:

Table 4 Detailed Time and Events Table for Day 10

Procedure		Day 10												Day 11	Day 12
	Pre-dose	0 min	5 min	15 min	30 min	45 min	1h	1.5h	2h	4h	6h	8h	12h	24h	48h
Dosing		X													
Vital Signs (BP and HR)	X		X	X		X		X		X		X	X	X	X
12-lead ECG	X			X		X		X		X		X	X	X	X
Blood PK sampling	X		X	X	X		X		X	X	X	X	X	X	X
Lab safety tests													X[1]		
SAE/AE/concurrent medication questioning	X														

1. Blood for lab tests will be taken after the blood sampling

Amended text:

Table 4 Detailed Time and Events Table for Day 10

Procedure		Day 10												Day 11	Day 12
	Pre-dose	0 min	5 min	15 min	30 min	45 min	1h	1.5h	2h	4h	6h	8h	12h	24h	48h
Dosing		X													
Vital Signs (BP and HR)	X		X	X		X		X		X		X	X	X	X
12-lead ECG	X			X		X		X		X		X	X	X	X
Blood PK sampling	X		X	X	X		X		X	X	X	X	X	X	X
Lab safety tests														X[1]	
SAE/AE/concurrent medication questioning	X														

1. Blood for lab tests will be taken after the blood PK sampling

Change #5

Change #5: To update the blood pressure in eligibility criteria.

Reason for change #5: To be consistent with the normal range in the healthy.

5.2.1 Inclusion criteria:

Original text:

4. Normal systolic (100-139mmHg) and diastolic (60-90mmHg) blood pressure at pre-study screening.

Amended text:

4. Normal systolic (90-139mmHg) and diastolic (60-89mmHg) blood pressure at pre-study screening.

Change #6

Change #6: To add X-ray into exclusion criteria.

Reason for change #6: To confirm the subject is not suffering from pulmonary disease of silent stage, e.g., Tuberculosis or cancer.

5.2.2 Exclusion criteria:

Original text:

None.

Amended text:

4. A chest X-ray or computed tomography (CT) scan that reveals evidence of clinically significant abnormalities. A chest X-ray must be taken at day -1 of the first treatment if a chest X-ray or CT scan is not available within 6 months prior to that day.

Change #7

Change #7: To add positive result of urine cotinine into eligibility criteria.

Reason for change #7: A subject denying current smoking may reach a positive result of urine cotinine because of second-hand smoking.

5.2.2 Exclusion criteria:

Original text:

None.

Amended text:

7. Positive result of urine cotinine test.

Change #8

Change #8: To change maximum total volume of blood sampling from 500 ml to 350 ml.

Reason for change #8: To meet requirement of site EC. The present volume has been confirmed enough for PK analysis by DMPK expert.

7. Study Assessments and Procedures

Original text:

No more than 500 mL of blood will be collected over the duration of the study, including any extra assessments that may be required.

Amended text:

No more than 350 mL of blood will be collected over the duration of the study, including any extra assessments that may be required.

Change #9

Change #9: To revise the position of ECG and vital signs.

Reason for change #9: To be consistent with both clinical practice and DB2114634 (an ongoing phase III study on Zephyr in China).

7.2 Safety: Vital Signs

Original text:

Subject will be required to rest in the semi-recumbent position at ~45 degrees for at least 5mins before each reading.

Amended text:

Subject will be required to rest in the sit position for at least 5mins before each reading.

7.2 Safety: Electrocardiogram (ECG)

Original text:

ECG measurements will be made (1-5mins apart for repeat measurements) with the subject in a semi-recumbent at 45 degrees having rested in this position for at least 5 mins before each reading.

Amended text:

ECG measurements will be made (1-5mins apart for repeat measurements) with the subject in a supine having rested in this position for at least 5 mins before each reading.

Change #10

Change #10: To update some details of lifestyle.

Reason for change #10: To provide both feasibility and quality.

8.2 Meals and Dietary Restrictions:

Original text:

Subjects must refrain from grapefruit or grapefruit juice containing products from 7 days pre-dose (Day 1) of the first treatment period until collection of the final PK blood sample.

Subjects will receive standardized meals during their in-house periods as described in the SPM.

Amended text:

Subjects must refrain from grapefruit or grapefruit juice containing products from 7 days pre-dose (Day 1) of the first treatment period until collection of the final PK blood sample. Subsequent meals and snacks will be standardized for all subjects during their in-site period.

8.3 Caffeine, Alcohol, and Tobacco

Original text:

Use of tobacco products is not allowed from the start of dosing and until after the final follow-up visit.

Amended text:

Use of tobacco products is not allowed from screening until after the final follow-up visit.

8.4 Activity:

Original text:

Subjects will abstain from strenuous exercise for 48 hours prior to each blood collection for clinical laboratory tests. Subjects may participate in light recreational activities during studies (e.g., watch television, read).

Amended text:

Subjects will abstain from strenuous exercise for 48 hours prior to each blood collection for clinical laboratory tests. Subjects may participate in light recreational activities during studies (e.g., watch television, read). Strong activities and long time bed rest should be avoided.

Change #11

Change #11: To reorganize the permitted and prohibited medications.

Reason for change #11: To keep the text smooth and clarified.

9. Concomitant Medications and Non-Drug Therapies:

Original text:

9.1Permitted Medications

Except for simple analgesics, over the counter preparations including vitamins, herbal remedies, and dietary supplements will not be permitted for 14 days before each study day, unless it is judged by the investigator not to compromise subject safety or influence the outcome of the study.

The Investigator (or designated study physician) must be informed as soon as possible about any medication taken from the time of screening until the end of the clinical phase of the study (follow-up visit).

All concomitant medications taken during the study will be recorded in the CRF. The minimum requirement is that drug name and the dates of administration are to be recorded.

9.2Prohibited Medications

Other than simple analgesics, such as paracetamol/acetaminophen, aspirin, ibuprofen, and et al , subjects must abstain from taking prescription or non-prescription drugs (including vitamins and dietary or herbal supplements), within 7 days (or 14 days if the drug is a potential enzyme inducer) or 5 half-lives (whichever is longer) prior to the first dose of study medication until completion of the follow-up visit, unless in the opinion of the Investigator and sponsor the medication will not interfere with the study.

9.3Non-Drug Therapies

Subjects must abstain from taking any vitamins, herbal and dietary supplements within 7 days (or 14 days if the drug is a potential enzyme inducer) or 5 half-lives (whichever is longer) prior to the first dose of study medication until completion of the follow-up visit, unless in the opinion of the Investigator and sponsor the medication will not interfere with the study. Strong activities and long time bed rest should be avoided.

Amended text:

9.1Permitted Medications

Subjects may take simple analgesics, such as paracetamol/acetaminophen, aspirin, ibuprofen, and et al, following the prescription of professional physician.

9.2Prohibited Medications

Other than simple analgesics, such as paracetamol/acetaminophen, aspirin, ibuprofen, and et al , subjects must abstain from taking prescription or non-prescription drugs (including vitamins and dietary or herbal supplements), within 7 days (or 14 days if the drug is a potential enzyme inducer) or 5 half-lives (whichever is longer) prior to the first dose of study medication until completion of the follow-up visit, unless in the opinion of the Investigator and sponsor the medication will not interfere with the study.

The Investigator (or designated study physician) must be informed as soon as possible about any medication taken from the time of screening until the end of the clinical phase of the study (follow-up visit).

All concomitant medications taken during the study will be recorded in the CRF. The minimum requirement is that drug name and the dates of administration are to be recorded.

9.3Non-Drug Therapies

The Investigator (or designated study physician) must be informed as soon as possible about any non-drug therapies from the time of screening until the end of the clinical phase of the study (follow-up visit).

Change #12

Change #12: To update SAE report tool from InForm to Fax.

Reason for change #12: InForm system will not be utilized in this study.

12.7 Prompt Reporting of SAEs to GSK:

Original text:

The primary mechanism for reporting SAEs to GSK will be the electronic data collection tool (e.g., InForm system). If the electronic system is unavailable for greater than 24 hours, the site will use the paper SAE data collection tool and fax it to the GSK Medical Monitor Dr [redacted]. Then the site will enter the serious adverse event data into the electronic system as soon as it becomes available.

After the study is completed at a given site, the electronic data collection tool (e.g., InForm system) will be taken off-line to prevent the entry of new data or changes to existing data. If a site receives a report of a new SAE from a study participant or receives updated data on a previously reported SAE after the electronic data collection tool has been taken off-line, the site can report this information on a paper SAE form or to their GSK protocol contact by telephone.

Amended text:

The primary mechanism for reporting SAEs to GSK will be using the paper SAE data collection tool and fax it to the GSK Medical Monitor Dr [redacted].

After the study is completed at a given site, if the site receives a report of a new SAE from a study participant or receives updated data on a previously reported SAE after the electronic data collection tool has been taken off-line, the site can report this information on a paper SAE form or to their GSK protocol contact by telephone.

Change #13

Change #13: To update the time and events tables.

Reason for change #13: To keep consistent with body text updated as above.

4.6 Time and Events Table:

Original text:

Table 2 Study Procedures

Procedure	Screening	day in each period											Follow-Up		
		Day -1	Day 1	Day 2	Day 3	Day 4	Day 5	Day 6	Day 7	Day 8	Day 9	Day 10	Day 11	Day 12	7-10 days after last dose
Admission to the unit		X[1]										X[2]			
Discharge from the unit			X[1]										X		
Training session		X													
Informed consent	X														
Medical history, Physical examination, height & weight[3]	X														X[4]
Serology (HIV, Hep B & C)	X														
Drugs of abuse screen	X														
Alcohol breath test and urine cotinine test	X	X													
Laboratory safety tests[5]	X	X[6]										X			X[7]
Vital signs (HR and BP)	X[8]	X	X	X	X	X	X	X	X	X	X	X	X	X	X
12-lead ECG	X[8]		X									X	X	X	X
Urine pregnancy test	X	X[6]										X[9]			
Review entry criteria	X	X													
Randomization		X[10]													
Dosing (AM)			X	X	X	X	X	X	X	X	X	X			
Blood PK sampling			X					X[11]	X[11]	X[11]	X[11]	X	X	X	
Unit visit[12]	X			X	X	X	X	X	X	X	X			X	
SAE/AE/concurrent medication questioning[13]	X														

1. On day -1 subjects will attend the unit and will be resident until all assessments completed at 8 hour post dosing on day 1.
2. On day 10, subjects will remain resident until 24 hour assessments are completed after the last dosing.
3. Height & body weight will be recorded at screening visit.
4. Physical examination only
5. clinical chemistry, haematology & urinalysis
6. These assessments are taken Day -1 in treatment 2 and 3.
7. Abnormal lab safety findings post-dosing should be followed up until the tests are normal, stable, no causal relationship with investigational product judged by investigators, or lost contact.
8. Three measurements are to be taken.
9. The test will be taken on Day 10 in the last treatment period.
10. Only on Day-1 in the first treatment period.
11. Blood PK sample will be collected only pre-dosing.
12. Day2-5, subjects visit unit for administration of investigational product under supervision of the investigators.
13. AEs and SAEs will be collected from the start of dosing with Investigational Product and until the follow-up visit. However, any SAEs related to study participation or a GSK concomitant medication will be recorded from the time a subject consents to participate in the study and until the follow-up visit.

Amended text:

<u>Detailed procedure and time, window please reference to SPM</u>.

Table 2 Study Procedures

**Procedure**	Screening	day in each period													Follow-Up
		Day -1	Day 1	Day 2	Day 3	Day 4	Day 5	Day 6	Day 7	Day 8	Day 9	Day 10	Day 11	Day 12	7-10 days after last dose
Admission to the unit		X[1]													
Discharge from the unit														X[1]	
Training session		X													
Informed consent	X														
Medical history, height & weight	X														
Radiography		X[2]													
Physical examination,	X[3]														X[4]
Serology (HIV, Hep B & C)	X														
Drugs of abuse screen	X														
Alcohol breath test and urine cotinine test	X	X													
Laboratory safety tests[5]	X	X[6]											X		X[7]
Vital signs (HR and BP)	X[8]	X	X	X	X	X	X	X	X	X	X	X	X	X	X

Procedure	Screening	day in each period													Follow-Up
		Day -1	Day 1	Day 2	Day 3	Day 4	Day 5	Day 6	Day 7	Day 8	Day 9	Day 10	Day 11	Day 12	7-10 days after last dose
12-lead ECG	X[8]		X									X	X	X	X
Urine pregnancy test	X	X[6]										X[9]			
Review entry criteria	X	X													
Randomization		X[10]													
Dosing (AM)			X	X	X	X	X	X	X	X	X	X			
Blood PK sampling			X					X[11]	X[11]	X[11]	X[11]	X	X	X	
Unit visit	X													X	
SAE/AE/concurrent medication questioning[12]	X														

1. On day -1 subjects will attend the unit and will be resident until all assessments completed on day12.
2. If a chest X-ray or CT scan is not available within 6 months prior to screening.
3. Physical examination means complete one.
4. Brief physical examination only.
5. Clinical chemistry, haematology & urinalysis.
6. These assessments are taken Day -1 in treatment period 2 and 3.
7. Abnormal lab safety findings post-dosing should be followed up until the tests are normal, stable, no causal relationship with investigational product judged by investigators, or lost contact.
8. Three measurements are to be taken.
9. The test will be taken on Day 10 in the last treatment period.
10. Only on Day-1 in the first treatment period.
11. Blood PK sample will be collected only pre-dosing.
12. AEs and SAEs will be collected from the start of dosing with Investigational Product and until the follow-up visit. However, any SAEs related to study participation or a GSK concomitant medication will be recorded from the time a subject consents to participate in the study and until the follow-up visit.

Change #14

Change #14: To update the description of investigational products.

Reason for change #14: To keep consistent with actual investigational products. Central supply is using single strip products for mono therapy, not the dual strip products.

4.1 Study Design/Schematic

Original text:

Table 1 Regimens of the study

Regimens	Treatments (inhaled once daily for 10 days)	
	NDPI Strip 1	NDPI Strip 2
A	UMEC 62.5µg	VI 25µg
B	UMEC 125µg	VI 25µg
C	UMEC 62.5µg	placebo
D	UMEC 125µg	placebo
E	placebo	VI 25µg

Amended text

Table 1 Regimens of the study

Regimens	Treatments (inhaled once daily for 10 days)	
	NDPI Strip 1	NDPI Strip 2
A	UMEC 62.5µg	VI 25µg
B	UMEC 125µg	VI 25µg
C	UMEC 62.5µg	N/A
D	UMEC 125µg	N/A
E	VI 25µg	N/A

N/A=Not Applicable

4.4 Investigational Product and Other Study Treatment Dosage/Administration

Original Text:

For individual UMEC treatment, the UMEC blister strip may contain approximately 62.5 or 125µg per blister of GSK573719, as the bromide salt. Second strip will be placebo.

For individual VI treatment, the VI blister strip will contain approximately 25µg per blister of GW642444 as the triphenylacetic acid salt. The other strip will be placebo.

		Investigational Product		
Product name:		UMEC	VI	UMEC and VI
Formulation description:	*1st* strip	GSK573719: micronised drug blended with lactose monohydrate and magnesium stearate[1]	GW642444 micronised drug blended with lactose monohydrate and magnesium stearate[2]	GSK573719: micronised drug blended with lactose monohydrate and magnesium stearate[1]
	2nd strip	Placebo: Lactose monohydrate and magnesium stearate	Placebo: Lactose monohydrate and magnesium stearate	GW642444: micronised drug blended with lactose monohydrate and magnesium stearate[2]
Dosage form:		Novel Dry Powder Inhaler	Novel Dry Powder Inhale	Novel Dry Powder Inhale
Unit dose strength(s)/Dosage level(s):		GSK573719 62.5µg or 125µg per blister	GW642444 25µg per blister	GSK573719 62.5µg or 125µg per blister, GW642444 25µg per blister
Route/ Administration/ Duration:		Inhaled, once daily single dose for 10 days	Inhaled, once daily single dose for 10 days	Inhaled, once daily single dose for 10 days
Dosing instructions:		62.5µg or 125µg once-daily for 10 days	25µg once-daily for 10 days	GSK573719 / GW642444 62.5µg/25µg or 125µg/25µg once-daily for 10 days
Physical description:		Dry off white powder	Dry off white powder	Dry off white powder
Device:		Novel Dry Powder Inhaler 30 doses per unit	Novel Dry Powder Inhaler 30 doses per unit	GSK573719:Novel Dry Powder Inhaler 30 doses per unit; GW642444: Novel Dry Powder Inhaler 30 doses per unit
Manufacturer/ source of procurement:		GSK	GSK	GSK

1. Magnesium stearate 0.6% w/w of total drug product
2. Magnesium stearate 1 % w/w of total drug product
3. The combination product (GSK573719 and GW642444) will be administered to the subject by using novel dry powder inhaler devices(NDPI)

Amended text

For individual UMEC treatment, the UMEC blister strip may contain approximately 62.5 or 125µg per blister of GSK573719, as the bromide salt.

For individual VI treatment, the VI blister strip will contain approximately 25µg per blister of GW642444 as the triphenylacetic acid salt.

		Investigational Product		
Product name:		UMEC	VI	UMEC and VI[3]
Formulation description:	*1st* strip	GSK573719: micronised drug blended with lactose monohydrate and magnesium stearate[1]	GW642444 micronised drug blended with lactose monohydrate and magnesium stearate[2]	GSK573719: micronised drug blended with lactose monohydrate and magnesium stearate[1]
	2nd strip	N/A	N/A	GW642444: micronised drug blended with lactose monohydrate and magnesium stearate[2]
Dosage form:		Novel Dry Powder Inhaler	Novel Dry Powder Inhale	Novel Dry Powder Inhale
Unit dose strength(s)/Dosage level(s):		GSK573719 62.5µg or 125µg per blister	GW642444 25µg per blister	GSK573719 62.5µg or 125µg per blister,<br>GW642444 25µg per blister
Route/ Administration/ Duration:		Inhaled, once daily single dose for 10 days	Inhaled, once daily single dose for 10 days	Inhaled, once daily single dose for 10 days
Dosing instructions:		62.5µg or 125µg once-daily for 10 days	25µg once-daily for 10 days	GSK573719 / GW642444 62.5µg/25µg or 125µg/25µg once-daily for 10 days
Physical description:		Dry off white powder	Dry off white powder	Dry off white powder
Device:		Novel Dry Powder Inhaler 30 doses per unit	Novel Dry Powder Inhaler 30 doses per unit	GSK573719:Novel Dry Powder Inhaler 30 doses per unit;<br>GW642444: Novel Dry Powder Inhaler 30 doses per unit
Manufacturer/ source of procurement:		GSK	GSK	GSK

N/A=Not Applicable

1. Magnesium stearate 0.6% w/w of total drug product
2. Magnesium stearate 1 % w/w of total drug product
3. The combination product (GSK573719 and GW642444) will be administered to the subject by using novel dry powder inhaler devices(NDPI)

Change #15

Change #15: To update report retention duration.

Reason for change #12: Meet the site's requirement, as well as GSK's policy.

14.6 Records Retention:

Original text:

GSK will inform the investigator of the time period for retaining these records to comply with all applicable regulatory requirements. The minimum retention time will meet the strictest standard applicable to that site for the study, as dictated by any institutional requirements or local laws or regulations, or GSK standards/procedures; otherwise, the retention period will default to 15 years.

Amended text:

According to ICH GCP and China GCP, all subject identification codes, subject files and Clinical Trial data and related documents will be kept by Institution in a secure place for a period of five (5) years after completion of the Clinical Trial. After the 5-year period, all documents kept by Institution will be transferred to the third party designated by GSK and kept in accordance with GSK policies. GSK will bear the archiving fee.
