# Supplementary material for: Pharmacokinetics and Tolerability of Inhaled Umeclidinium and Vilanterol Alone and in Combination in Healthy Chinese Subjects: A Randomized, Open-Label, Crossover Trial
Source: PLoS One. 2015 Mar 27;10(3):e0121264. doi: 10.1371/journal.pone.0121264 (PMC4376748; doi:10.1371/journal.pone.0121264)
Supplement: S1 Supporting Information — (DOC) [file pone.0121264.s003.doc]

# SUPPORTING INFORMATION S1

## Methods

### Inclusion and exclusion criteria

Eligible subjects were: healthy males or females of Chinese heritage; 18−45 years of age; had a body weight ≥50 kg; had a body mass index of 19−24 kg/m^2^; had normal systolic (90−139 mmHg) and diastolic (60−89 mmHg) blood pressure; were current non-smokers; had not used any tobacco products in the 6-month period preceding the screening visit; had a smoking history ≤10 pack years; had no significant abnormality on 12-lead electrocardiogram; had a QTcF interval <450 msec; had aspartate aminotransferase, alanine aminotransferase, and total-bilirubin ≤1.5 x upper limit of normal; had no significant clinical abnormality on other laboratory tests; were capable of giving written informed consent (including compliance with the requirements and restrictions listed in the consent form); and could use the inhalation device satisfactorily.

Exclusion criteria included: childbearing potential (unless the subject abstained, was sexually inactive, or used contraception methods with a failure rate of <1%); any significant medical condition that according to the investigator would prevent the patient from carrying out the study procedures safely; a history of mental, cardiac, renal, hepatic, significant gastrointestinal, or respiratory disease as judged by the investigator; a history of breathing problems (ie history of asthmatic symptomatology, unless asthma in childhood that had resolved and no longer required maintenance therapy; a chest X-ray (taken at Day -1 of the first treatment if a chest X-ray or computed tomography scan was not available within 6 months prior to that day) or computed tomography scan that revealed evidence of clinically-significant abnormalities; a history of sensitivity to heparin, heparin-induced thrombocytopenia, or sensitivity to any of the study medications, or components thereof; known allergy or hypersensitivity to milk protein or the excipients lactose monohydrate and magnesium stearate; a history of drug or other allergy that, in the opinion of the investigator or GSK Medical Monitor, contraindicated their participation; use of prescription or non-prescription drugs, including CYP3A/PGP inhibitor, vitamins, herbal and dietary supplements (including St John’s Wort) within 7 days (or 14 days if the drug is a potential enzyme inducer) or 5 half-lives (whichever is longer) prior to the first dose of study medication, unless in the opinion of the Investigator and Sponsor the medication was not expected to interfere with the study procedures or compromise subject safety; a positive urine cotinine test; a history of cholecystectomy or biliary tract disease; a significant clinical history of or current glaucoma; a significant clinical history of or current prostatic hypertrophy; a positive pre-study drug screen (a minimum list of drugs that were screened for included amphetamines, barbiturates, cocaine, opiates and benzodiazepines); detection of alcohol pre-dose or during the study; a history of regular alcohol consumption within 3 months of the study defined as abuse of an average weekly intake >21 units or an average daily intake >3 units in males, or an average weekly intake >14 units or an average daily intake >2 units in females; pregnancy (or planned pregnancy and lactation); participation in a clinical trial and use of an investigational product within the following time period prior to the first dosing day in the current study: 30 days, 5 half-lives or twice the duration of the biological effect of the investigational product (whichever is longer); blood donation or blood sampling as a study subject within 3 months preceding the first dose of study drug and blood donation during the entire study in excess of 500 mL; positive pre-study Hepatitis B surface antigen or positive Hepatitis C antibody result within 3 months of screening; a positive test for human immunodeficiency virus antibodies; unwillingness or inability to follow the procedures outlined in the protocol; or mental or legal incapacity.)

|  |  |  |
| --- | --- | --- |
|  |  |  |
|  |  |  |
|  |  |  |
|  |  |  |

|  |  |  |  |
| --- | --- | --- | --- |
|  |  |  |  |
|  |  |  |
|  |  |  |
|  |  |  |
|  |  |  |  |
|  |  |  |
|  |  |  |
|  |  |  |

|  |  |  |  |
| --- | --- | --- | --- |
|  |  |  |  |
|  |  |  |
|  |  |  |
|  |  |  |  |
|  |  |  |
|  |  |  |
