# Supplementary material for: Pharmacokinetics and Tolerability of Inhaled Umeclidinium and Vilanterol Alone and in Combination in Healthy Chinese Subjects: A Randomized, Open-Label, Crossover Trial
Source: PLoS One. 2015 Mar 27;10(3):e0121264. doi: 10.1371/journal.pone.0121264 (PMC4376748; doi:10.1371/journal.pone.0121264)
Supplement: S1 Table — A: UMEC/VI 62.5/25 μg; B: UMEC/VI UMEC/VI 125/25 μg; C: UMEC 62.5 μg; D: UMEC 125 μg; E: VI 25 μg. (DOCX) [file pone.0121264.s004.docx]

## Table S1. Treatment sequences for 20 subjects in this balanced, incomplete-block, three-way crossover study

| **Sequence** | **Period 1** | **Period 2** | **Period 3** |
| --- | --- | --- | --- |
| 1 | A | E | D |
| 2 | B | A | E |
| 3 | C | B | A |
| 4 | D | C | B |
| 5 | E | D | C |
| 6 | A | B | C |
| 7 | B | C | D |
| 8 | C | D | E |
| 9 | D | E | A |
| 10 | E | A | B |
| 11 | A | D | B |
| 12 | B | E | C |
| 13 | C | A | D |
| 14 | D | B | E |
| 15 | E | C | A |
| 16 | A | C | E |
| 17 | B | D | A |
| 18 | C | E | B |
| 19 | D | A | C |
| 20 | E | B | D |

A: UMEC/VI 62.5/25 µg; B: UMEC/VI UMEC/VI 125/25 µg; C: UMEC 62.5 µg; D: UMEC 125 µg; E: VI 25 µg
