# Supplementary material for: Pharmacokinetics and Tolerability of Inhaled Umeclidinium and Vilanterol Alone and in Combination in Healthy Chinese Subjects: A Randomized, Open-Label, Crossover Trial
Source: PLoS One. 2015 Mar 27;10(3):e0121264. doi: 10.1371/journal.pone.0121264 (PMC4376748; doi:10.1371/journal.pone.0121264)
Supplement: S2 Table — A mixed model with treatment, day and treatment-by-day interaction as covariates was used in the steady-state evaluation.*Day 11 was the 24-h post-dose concentration on Day 10. CI, confidence interval; PK, pharmacokinetic; UMEC, umeclidinium; VI, vilanterol. (DOC) [file pone.0121264.s005.doc]

## Table S2. Statistical analysis of UMEC PK parameters to determine attainment of steady-state (based on visual observation of individual trough concentrations on Days 6–11^*^)

| **Treatment** | **Slope estimate** | **90% CI of slope** |
| --- | --- | --- |
| UMEC/VI 62.5/25 µg | -0.025 | -0.053, 0.003 |
| UMEC 62.5 µg | 0.021 | -0.004, 0.045 |
| UMEC/VI 125/25 µg | 0.062 | 0.046, 0.077 |
| UMEC 125 µg | 0.075 | 0.060, 0.090 |

^*^Day 11 was the 24-h post-dose concentration on Day 10. CI, confidence interval; PK, pharmacokinetic; UMEC, umeclidinium; VI, vilanterol.
