# Supplementary material for: Pharmacokinetics and Tolerability of Inhaled Umeclidinium and Vilanterol Alone and in Combination in Healthy Chinese Subjects: A Randomized, Open-Label, Crossover Trial
Source: PLoS One. 2015 Mar 27;10(3):e0121264. doi: 10.1371/journal.pone.0121264 (PMC4376748; doi:10.1371/journal.pone.0121264)
Supplement: S3 Table — A mixed model fitted with day as a fixed effect and subject as the random effect was used in the accumulation assessment (by treatment). AUC(0–2), area under the concentration-time curve from time zero to 2 h; CI, confidence interval; Cmax, maximum plasma concentration; PK, pharmacokinetic; UMEC, umeclidinium; VI, vilanterol. (DOC) [file pone.0121264.s006.doc]

**Table S3. Statistical analysis of UMEC PK parameters to assess accumulation: ratio of geometric means (Day 10/Day 1)**

| **Parameter** | **Treatment** | **Ratio of geometric means** | **90% CI of the ratio** |
| --- | --- | --- | --- |
| AUC_(0–2)_, pg.hr/mL | UMEC/VI 62.5/25 µg | 1.31 | 1.16, 1.49 |
|  | UMEC/VI 125/25 µg | 1.43 | 1.34, 1.53 |
|  | UMEC 62.5 µg | 1.19 | 0.89, 1.59 |
|  | UMEC 125 µg | 1.59 | 1.40, 1.81 |
| C_max_ (pg/mL) | UMEC/VI 62.5/25 µg | 1.17, | 1.02, 1.34 |
|  | UMEC/VI 125/25 µg | 1.16 | 1.08, 1.25 |
|  | UMEC 62.5 µg | 1.11 | 0.79, 1.55 |
|  | UMEC 125 µg | 1.34 | 1.14, 1.56 |

AUC_(0–2)_, area under the concentration-time curve from time zero to 2 h; CI, confidence interval;
C_max_, maximum plasma concentration; PK, pharmacokinetic; UMEC, umeclidinium; VI, vilanterol.
