# Supplementary material for: Pharmacokinetics and Tolerability of Inhaled Umeclidinium and Vilanterol Alone and in Combination in Healthy Chinese Subjects: A Randomized, Open-Label, Crossover Trial
Source: PLoS One. 2015 Mar 27;10(3):e0121264. doi: 10.1371/journal.pone.0121264 (PMC4376748; doi:10.1371/journal.pone.0121264)
Supplement: S4 Table — A mixed model fitted with day as the fixed effect and subjects as the random effect was used in the accumulation assessment (by treatment). AUC(0–2), area under the concentration-time curve from time zero to 2 h; CI, confidence interval; Cmax, maximum plasma concentration; PK, pharmacokinetic; UMEC, umeclidinium; VI, vilanterol. (DOC) [file pone.0121264.s007.doc]

**Table S4. Statistical analysis of VI PK parameters to assess accumulation**: ratio of geometric means (Day 10/Day 1)

| **Parameter** | **Treatment** | **Ratio of geometric means** | **90% CI of the ratio** |
| --- | --- | --- | --- |
| AUC_(0–2)_, pg.hr/mL | UMEC/VI 62.5/25 µg | 1.17 | 1.03, 1.32 |
|  | UMEC/VI 125/25 µg | 1.30 | 1.04, 1.61 |
|  | VI 25 µg | 1.43 | 1.20, 1.70 |
| C_max_ (pg/mL) | UMEC/VI 62.5/25 µg | 1.25 | 1.04, 1.49 |
|  | UMEC/VI 125/25 µg | 1.66 | 1.03, 2.66 |
|  | VI 25 µg | 1.41 | 1.15, 1.72 |

AUC_(0–2)_, area under the concentration-time curve from time zero to 2 h; CI, confidence interval;
C_max_, maximum plasma concentration; PK, pharmacokinetic; UMEC, umeclidinium; VI, vilanterol.
